# Dual‐Functional and Low‐Toxicity ZnO Nanoparticles From Pomegranate Peel: Impact of Synthesis Parameters on Photocatalytic and Supercapacitive Performance

**DOI:** 10.1002/open.70209

**Published:** 2026-04-21

**Authors:** Roumaissa Djafarou, Ouarda Brahmia, Naima Benchikha, Fatma Kılıç Dokan, Ertugrul Sahmetlioglu, Ayomide Victor Atoki, Mohammed Messaoudi

**Affiliations:** ^1^ Laboratoire des Techniques Innovantes de Préservation de l’Environnement Université de Constantine 1 Constantine Algeria; ^2^ Laboratory of Applied Chemistry and Environment (LCAE) Department of Chemistry Faculty of Exact Sciences University of Hamma Lakhdar El‐Oued Algeria; ^3^ Department of Chemistry and Chemical Processing Technologies Mustafa Çıkrıkcıoglu Vocational School Kayseri University Kayseri Turkey; ^4^ Department of Basic Sciences of Engineering Kayseri University Kayseri Turkey; ^5^ Department of Biochemistry Kampala International University Ishaka Uganda; ^6^ Laboratory of Research on Bioactive Products and Biomass Valorization Department of Chemistry Higher Normal School of Kouba (ENS) Algiers Algeria

**Keywords:** bifunctional performance, ecotoxicity, energy storage, green synthesis, photocatalysis, pomegranate peel extract, supercapacitors, waste valorization, zinc oxide nanoparticles

## Abstract

The development of eco‐efficient multifunctional nanomaterials is crucial for environmental remediation and energy storage. This study systematically investigated the impact of synthesis temperature (25°C vs. 80°C) and zinc acetate concentration (0.5–2 M) on pomegranate peel extract‐mediated ZnO nanoparticles. Physicochemical characterization revealed that ambient synthesis (25°C) at 2 M (sample A2) produced an interconnected hierarchical architecture with a specific surface area (SSA_BET_) of 12.1 m^2^/g, a zeta potential of −28.6 mV, and a narrow bandgap of 2.744 eV. Consequently, A2 exhibited superior bifunctional performance, achieving a photocatalytic rate constant (*k*_app_) of 0.065 min^−1^ for the degradation of Methylene Blue (10 ppm) and a specific capacitance of 208 F g^−1^ at 1 A g^−1^. Crucially, electrochemical impedance spectroscopy confirmed that the interconnected framework of A2 provided a negligible charge–transfer resistance (*R*_p_ = 0.0043 Ω), facilitating efficient charge transport compared to the resistive, fragmented morphologies produced at 80°C (*R*_p_ = 3069.64 Ω). Furthermore, A2 demonstrated low acute ecotoxicity toward *Artemia salina* (LC_50_ = 4842 μg/mL). These findings provide a data‐driven framework for the low‐energy synthesis of high‐performance, sustainable ZnO nanomaterials.

## Graphical abstract

The synthesis of ZnO nanoparticles (NPs) using pomegranate peel extract at ambient temperature represents a significant advancement in the development of sustainable dual‐functional materials. These NPs demonstrated high photocatalytic efficiency in the degradation of organic pollutants and exhibited significant supercapacitive energy storage capabilities, along with a substantially improved ecotoxicity profile. Consequently, they have emerged as promising candidates for integrated environmental remediation and green energy storage systems. This eco‐friendly synthesis method provides a scalable approach for developing advanced materials to address concurrent environmental and energy challenges.

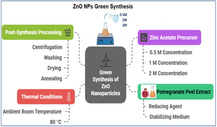

## 1 Introduction

The increasing global demand for environmental remediation and sustainable energy storage has spurred extensive research on advanced multifunctional nanomaterials [1, 2, 3]. Zinc oxide (ZnO) nanoparticles (NPs) are particularly noteworthy because of their diverse physicochemical properties, including their inherent photocatalytic ability to degrade organic pollutants and promising electrochemical characteristics suitable for supercapacitors [4, 5]. However, traditional ZnO synthesis methods often involve hazardous chemicals, are energy‐intensive, and entail complex procedures, thereby posing significant environmental and scalability challenges [6, 7, 8]. Biogenic synthesis methods, particularly those utilizing plant extracts, offer eco‐friendly, cost‐effective, and sustainable alternatives by employing biocompatible phytochemicals as reducing and capping agents [9, 10, 11]. Pomegranate peel extract (PPE), an agricultural by‐product rich in polyphenolic compounds such as ellagic acid and punicalagins, has been demonstrated to be an effective green reagent for ZnO synthesis, promoting circular economy principles through waste valorization [12, 13, 14]. The functional efficacy of ZnO is critically dependent on its adjustable physicochemical properties, such as particle size, shape, specific surface area, porosity, crystallinity (predominantly the wurtzite phase), surface charge (zeta potential (ZP)), and the nature and concentration of intrinsic defects, specifically oxygen vacancies (*V*_O_) [15, 16, 17]. These features are intricately controlled by the synthesis conditions, with the reaction temperature and precursor concentration being pivotal factors that govern nucleation rates, crystal growth kinetics, and defect formation [18, 19, 20]. Our previous studies on green‐synthesized ZnO, particularly concerning the dual‐functional potential of starch‐derived ZnO NPs [21], underscore the promise of biogenic methods. However, that study also highlighted the necessity for a deeper understanding of how fundamental synthesis variables, when combined with different natural precursors, dictate the resulting properties and optimize distinct functionalities. Building on these insights, the current study focused on PPE as a unique, readily available biogenic source to systematically explore the impact of key synthesis parameters on PPE‐derived ZnO to enhance its multifunctional performance. Although numerous studies have investigated PPE‐mediated ZnO synthesis, they typically focus on single‐application optimization, such as photocatalysis [22, 23], and less frequently on energy storage [24, 25]. Despite these advancements, a comprehensive understanding of how the synthesis temperature and precursor concentration synergistically influence the physicochemical properties of PPE‐derived ZnO for dual applications remains lacking. Furthermore, the specific interplay between ambient and higher temperatures in determining the ideal precursor concentration for simultaneous optimization has not been well established. Filling this knowledge gap is essential for the rational design of multifunctional biosynthesized ZnO nanomaterials intended for integrated environmental and energy applications. In response to existing challenges in sustainable nanotechnology, this study aimed to optimize the bifunctional performance of ZnO NPs by systematically investigating the synergistic effects of synthesis temperature (25°C vs. 80°C) and zinc acetate precursor concentration (0.5–2 M) utilizing PPE. This research marks a significant advancement in green manufacturing by demonstrating that superior functional efficiency can be achieved at a strictly ambient temperature (25°C), representing a 55°C reduction in thermal requirements compared to conventional biogenic protocols, achieved without compromising material performance. A central scientific contribution of this work is the identification of a “connectivity versus surface area” paradox; we demonstrated that morphological interconnectivity is a more critical determinant of charge‐carrier kinetics than absolute surface area. Notably, the ambient‐synthesized sample A2 consistently outperformed the fragmented 80°C variants (e.g., T2), despite possessing a 38% lower nominal surface area. This structural advantage was quantitatively validated through electrochemical impedance spectroscopy (EIS), which revealed a near‐zero polarization resistance (*R*_p_ = 0.0043 Ω) for A2, suggesting a seamless biogenic–inorganic interface plausibly mediated by carboxylate “molecular bridges” preserved under ambient conditions. By achieving a simultaneous optimization of photocatalytic efficiency (*k*_app_ = 0.065 min^−1^) and pseudocapacitance (208 Fg^−1^), while integrating a comprehensive ecotoxicological safety profile (LC_50_ = 4842 μg/mL), this work establishes a robust data‐driven framework for the sustainable design of multifunctional nanomaterials.

## 2 Materials and Methods

### 2.1 Materials

Zinc acetate dihydrate (C_4_H_6_O_4_Zn·2H_2_O, 99%, Honeywell Fluka) was used as the zinc precursor, and distilled water was used as the solvent. Methylene blue MB) (Sigma–Aldrich) was used as the model pollutant for the photocatalytic degradation assays, and isopropanol (Sigma–Aldrich) was used as a radical scavenger. For electrode fabrication, polyvinylidene fluoride (PVDF), acetylene black, and N‐methyl‐2‐pyrrolidone (NMP) were purchased from Sigma–Aldrich. Potassium hydroxide (KOH) and hydrochloric acid (HCl, 37%) were supplied by Merck. All reagents were of analytical grade and used without further purification.

### 2.2 Synthesis Protocols

#### 2.2.1 PPE Preparation

Pomegranate peels (Punica granatum, Algerian variety) were shade‐dried at an ambient temperature of 25°C, followed by pulverization into a fine powder. Ultrasound‐assisted aqueous extraction was performed using distilled water as the solvent to selectively extract water‐soluble bioactive compounds, particularly polyphenols and flavonoids. These compounds were well‐documented for their potent reducing and stabilizing properties in the green synthesis of NPs. Following extraction, the crude mixture was filtered and centrifuged at 7000 rpm for 5 min. The resulting supernatant was stored at 4°C for use in the biosynthesis of ZnO NPs.

#### 2.2.2 Green Synthesis of ZnO NPs

**SCHEME 1:**
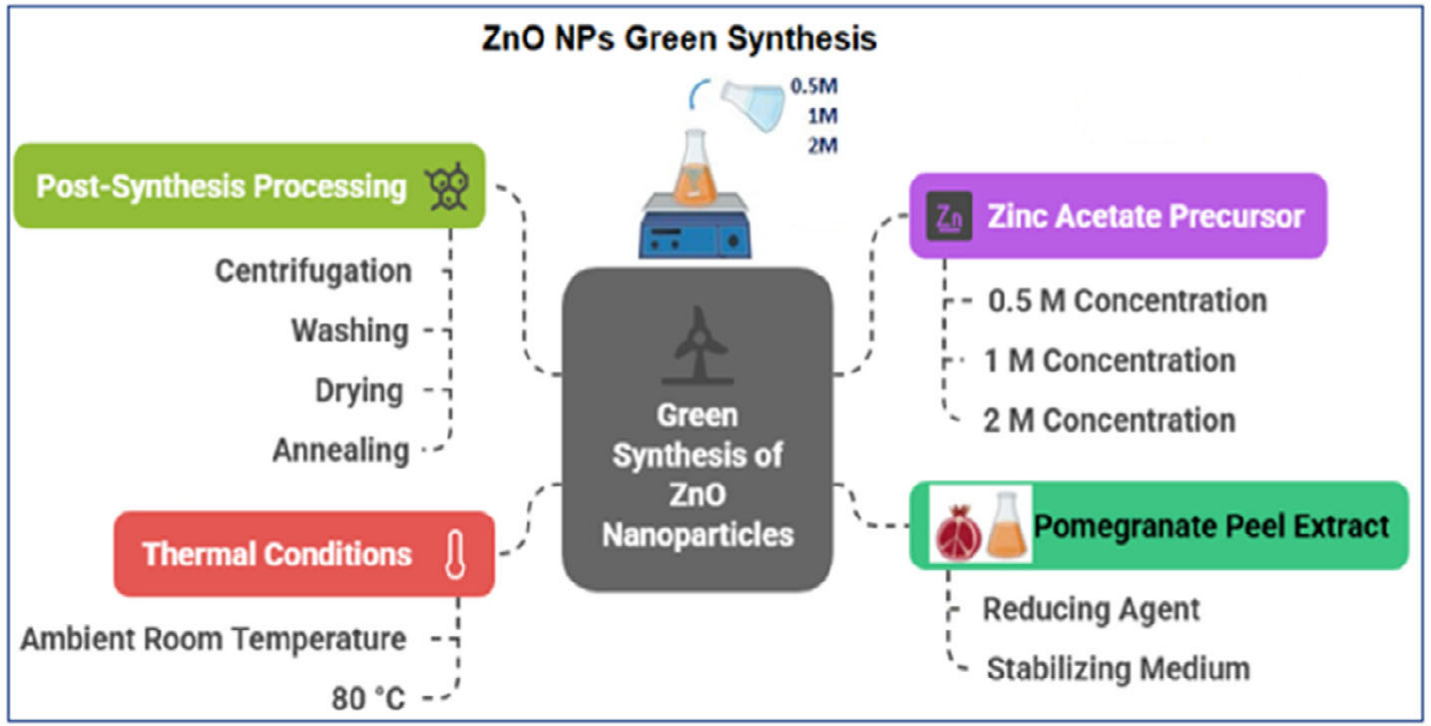
Schematic illustration of the green sol–gel synthesis protocol for ZnO NPs utilizing PPE as a dual‐functional agent.

In this study, ZnO NPs were synthesized via a green sol–gel method employing PPE as a dual‐functional reducing and stabilizing agent (Scheme 1). To ensure high reproducibility, zinc acetate precursor solutions (0.5, 1, and 2 M) were combined with the aqueous PPE solution at a precise volume ratio of 1:10 (v/v), specifically by adding 10 mL of zinc precursor to 100 mL of PPE extract. This process was conducted under vigorous magnetic stirring. The natural pH of the reaction mixture was monitored and recorded at 5.4 ± 0.2, and this value was strictly maintained without any external chemical titration (acidic or basic) to preserve a purely biogenic synthesis environment. The reaction mixtures were then subjected to an overnight aging process under two distinct thermal conditions: one set was maintained at ambient temperature (~25°C), and the other at a controlled temperature of 80°C. The formation of ZnO NPs was visually identified by the appearance of a characteristic yellowish precipitate. After aging, the solid phase was isolated via centrifugation at 6000 rpm for 10 min. To remove unreacted precursors and organic residues, the collected precipitate was washed thrice with distilled water. The washed product was then dried in an oven at 80°C for 24 h to eliminate residual moisture. A final annealing step was conducted at 600°C for 2 h for all samples under atmospheric conditions to maximize crystallinity and ensure the formation of a pure ZnO phase. The ZnO NPs synthesized at ambient temperature were designated as A0.5, A1, and A2 (corresponding to 0.5, 1, and 2 M precursor concentrations, respectively), whereas those synthesized at 80°C were designated as T0.5, T1, and T2.

### 2.3 Characterization Methods

The physicochemical properties of the synthesized ZnO NPs were characterized using advanced analytical techniques. The crystalline structure and phase purity were determined by X‐ray diffraction (XRD) using a D8 ADVANCE diffractometer (Bruker, Massachusetts, USA) with Cu Kα radiation (*λ* = 1.5406 Å). The morphology and elemental composition were analyzed via field‐emission scanning electron microscopy (FESEM) and scanning transmission electron microscopy (STEM) on a Gemini 550 microscope (Zeiss, Oberkochen, Germany) equipped with energy dispersive X‐ray spectroscopy (EDS). The optical properties and bandgap were investigated using UV–visible spectroscopy (Shimadzu 1800, Kyoto, Japan), and the chemical bonding was examined via Fourier transform infrared (FTIR) spectroscopy (Spotlight 400, PerkinElmer, Waltham, MA, USA). The surface characteristics were determined through N_2_ adsorption–desorption isotherms at 77 K using a Gemini VII analyzer (Micromeritics, Mönchengladbach, Germany), and the specific surface area (SSA_BET_) and pore size were calculated using the Brunauer–Emmett–Teller (BET) and Barrett–Joyner–Halenda (BJH) models, respectively. Finally, the surface charge and colloidal stability were assessed by measuring the ZP using a Malvern Zetasizer Nano ZS90.

### 2.4 Photocatalytic Activity Evaluation

#### 2.4.1 Experimental Setup

A custom‐designed photoreactor was used to perform controlled photocatalytic experiments. The experimental setup comprised a 150 mL cylindrical quartz reaction vessel and a 15 W low‐pressure mercury UV lamp (*λ*_max_ = 365 nm) for catalyst activation. MB, a cationic dye that serves as a model for industrial pollutants such as textile effluents, was employed as the model pollutant. Its strong absorption in the visible region and well‐documented degradation kinetics facilitate real‐time efficiency monitoring using UV–vis spectrophotometry. The environmental prevalence and ecological significance of MB further justify its use in evaluating practical water treatment applications.

#### 2.4.2 Degradation Procedure

The photocatalytic efficacy of the six synthesized ZnO variants (A0.5, A1, and A2, synthesized at 25°C, and T0.5, T1, and T2, synthesized at 80°C) was assessed in relation to the degradation of MB under UV irradiation. For each experimental trial, 0.1 g of the ZnO catalyst was dispersed in 100 mL of a 10 mg/L aqueous MB solution, corresponding to a catalyst loading of 1 g/L. The suspension was magnetically stirred in the dark for 45 min to achieve adsorption–desorption equilibrium. Following this equilibration period, an initial sample was collected to establish the baseline concentration (*C*_0_) prior to the commencement of irradiation, during which the dark adsorption capacity was determined to be 8% under initial, unadjusted, neutral pH conditions. Under continuous UV light exposure, 3 mL aliquots were extracted at predetermined intervals. These samples were centrifuged at 7000 rpm for 10 min to remove the catalyst particles, and the residual MB concentration (*C*_t_) in the supernatant was quantified using a UV–vis spectrophotometer at λ_max_ = 652 nm. The degradation efficiency (%) was calculated using Equation (1).

(1) $$\text{Degradation}\;\left(\%\right)\;=\;\frac{\left(C_{0}-C_{t}\right)}{C_{0}}\times 100$$

The reaction kinetics were analyzed using the pseudo‐first‐order model, as shown in Equation (2):

(2) $$\ln\frac{C_{0}}{C_{t}}=\;k_{\mathrm{app}}\;\times t$$

where *k*_app_ is the apparent pseudo‐first‐order rate constant (min^−1^), and t is the irradiation time (min). All photocatalytic experiments were conducted in triplicate to ensure reproducibility and statistical reliability.

#### 2.4.3 Catalyst Recyclability

The ZnO catalyst, designated as A2 and identified as the most effective in the parametric studies, was subjected to four consecutive degradation cycles to evaluate its stability and reusability. After each degradation cycle, the catalyst was recovered by filtration, washed with distilled water, dried at 60°C for 6 h, and subsequently reused under identical conditions. The degradation kinetics, represented as *C*/*C*_0_ versus irradiation time, and the retention of efficiency across cycles were assessed to determine any decline in the performance.

### 2.5 Electrochemical Evaluation

#### 2.5.1 Electrode Preparation

During the electrode fabrication process, a slurry was prepared by dispersing the active material (ZnO, 80 wt%), conductive agent (acetylene black, 15 wt%), and binder (PVDF, 5 wt%) in the NMP solvent. This mixture was subsequently applied to 1 cm^2^ sections of a nickel foam substrate. The drying process at 80°C facilitated the removal of NMP, resulting in electrodes with a controlled active material mass of approximately 5 mg, thereby ensuring reliable electrochemical comparison.

#### 2.5.2 Electrochemical Characterization

Electrochemical measurements were conducted using a GAMRY Reference 3000 potentiostat configured with a standard three‐electrode setup. The ZnO material, prepared and coated on nickel foam, functioned as the working electrode, with a platinum foil (1 cm × 1 cm) as the counter electrode and a saturated Ag/AgCl reference electrode. All experiments were performed using a 2 M KOH aqueous electrolyte. The performance evaluation included cyclic voltammetry (CV), galvanostatic charge–discharge (GCD), and EIS measurements. CV scans were performed at rates ranging from 10 to 100 mV/s within a potential window of −0.2 to 0.7 V (vs. Ag/AgCl). GCD cycles were conducted at current densities ranging from 1 to 10 A/g. EIS spectra were recorded from 10 kHz to 0.01 Hz with an amplitude of 10 mV.

#### 2.5.3 Performance Calculations

The specific capacitance (C, F/g), specific energy density (E, Wh/kg), and specific power density (P, W/kg) were calculated from galvanostatic discharge data. Specific capacitance was calculated from the slope of the discharge curve using Equation (3):

(3) $$\;C=\;\frac{I\times \;\text{Δ}t}{m\times \;\text{Δ}V}$$

where *I* is the applied discharge current (A), δ*t* is the discharge duration (s), *m* is the mass of active material (g), and δ*V* is the potential window (*V*) during discharge.

The specific energy density (*E*) and specific power density (*P*) were computed using Equations (4) and (5), respectively:

(4) $$E=\frac{C\times \;\text{Δ}V^{2}}{2\;\times \;3.6}$$

(5) $$P=\;\frac{3600\;\times E}{\text{Δ}t}$$

### 2.6 In Vitro Ecotoxicity Assessment Using Brine Shrimp Lethality Assay (BSLA)

The in vitro ecotoxicity of the synthesized ZnO‐A2 NPs, identified as the most active for both photocatalytic and supercapacitive efficacy, was evaluated using the BSLA following a protocol adapted from Nookala Supraja et al. [26]. Briefly, *Artemia salina* cysts (1 g) were hatched in 1 L of filtered seawater (0.45 µm filter) within a hatching tank under continuous aeration and constant illumination and maintained at 25°C–28°C for 48 h to obtain nauplii (larvae). Test tubes were prepared in triplicate for each test concentration. Each tube contained 2 mL of filtered seawater and 2 mL of an aqueous ZnO‐A2 NPs suspension (prepared by sonication in distilled water to ensure dispersion), achieving final exposure concentrations ranging from 125 to 4000 µg/mL. The negative control group, run in triplicate, contained 2 mL of filtered seawater and 2 mL of distilled water. Ten freshly hatched Artemia salina nauplii were carefully transferred using a pipette to each test tube. The tubes were then incubated under the same temperature (25°C–28°C) and illumination conditions used for the hatching process. Following a 24 h incubation period, the viability of nauplii in each tube was assessed via visual inspection. The number of surviving nauplii (Ns) was recorded for each replicate, and the percentage mortality (M%) was calculated using Equation (6), as follows:

(6) $$\;\text{M\%}=\;\frac{\left(N_{t}-N_{s}\right)}{N_{t}}\times 100$$

where *N*_t_ represents the initial quantity of nauplii, set at 10, and *N*_s_ denotes the number of nauplii that survived after a 24 h period. The median lethal concentration (LC_50_), which is the concentration statistically estimated to result in 50% mortality within the exposed population, was derived from the obtained concentration‐response data. The analysis was conducted using Finney's probit analysis method, implemented through SPSS software (Version 26), and included the computation of 95% confidence intervals (CI).

### 2.7 Data Analysis

Statistical analyses were conducted using Origin Pro software (version 2018; OriginLab Corporation, Northampton, MA, USA). The threshold for statistical significance was set at *p* < 0.05. Data are reported as mean ± standard deviation (SD) from three independent measurements for all experimental assays, including photocatalytic degradation, electrochemical performance, and ecotoxicity evaluation.

## 3 Results and Discussion

### 3.1 FTIR Spectroscopy

**FIGURE 1:**
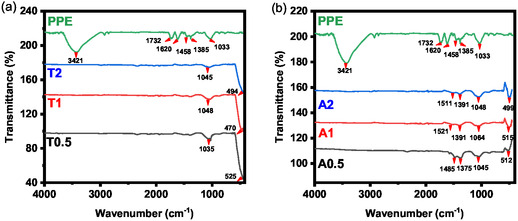
FTIR spectra of PPE and the ZnO samples synthesized at (a) elevated temperature (80°C), (b) at ambient temperature (25°C).

FTIR spectroscopy was employed to elucidate the surface functional groups of the PPE and to confirm the formation and purity of the synthesized ZnO NPs. The resulting spectra are presented in Figure 1. The FTIR spectrum of PPE reveals a broad and intense absorption band centered at approximately 3421 cm^−1^, indicative of O–H stretching vibrations [27, 28]. This band is attributed to the hydroxyl groups prevalent in polyphenolic compounds, alcohols, and carboxylic acids within the extract, as well as the associated water molecules. The distinct peak at 1732 cm^−1^ is associated with C═O stretching vibrations, likely originating from the carboxylic acid or amide functionalities present in PPE [27]. Additionally, the band at 1620 cm^−1^ can be ascribed to the C═C stretching vibrations of the aromatic rings or conjugated carbonyl systems within the phyto‐constituents of the extract [28]. Further bands observed between approximately 1521 and 1458 cm^−1^ may correspond to N─H bending vibrations or aromatic C═C skeletal vibrations [29, 30], while vibrational modes near 1391−1375 cm^−1^ are indicative of C─H bending [29, 31]. Strong absorptions in the 1064−1033 cm^−1^ region are characteristic of C─O stretching vibrations from alcohols, ethers, and ester groups within the organic matrix of the extract [30, 32].

Notably, all ZnO NPs samples exhibited prominent bands within the range of approximately 470–525 cm^−1^ (Figure 1a,b), corresponding to the characteristic Zn─O stretching vibrations. These bands confirmed the successful formation of the ZnO crystal lattice [33, 34, 35].

Upon the formation of ZnO NPs, significant spectral changes were observed compared to the PPE spectrum. The characteristic hydroxyl (approximately 3421 cm^−1^) and carbonyl (approximately 1732 cm^−1^) groups, which were prominent in the extract, were absent in all ZnO NP spectra. This observation strongly suggests the effective removal of these organic functional groups during the nanoparticle synthesis and purification processes, indicating their role as capping or reducing agents in green synthesis [36]. The disappearance of the N─H and C─H related bands, along with a significant reduction in the C─O stretching band (e.g., around 1033–1064 cm^−1^) in the T series spectra (Figure 1a), further substantiates the advanced transformation of organic biomolecules from the nanoparticle surface, potentially facilitated by thermal effects, leaving only minor oxygenated fragments. In contrast, the A‐series samples (Figure 1b) exhibited residual bands at ≈1485–1521 cm^−1^ ^1^ and 1375–1391 cm^−1^, corresponding to the stretching vibrations of carboxylate groups (COO^‐^) derived from organic acid residues. These surface‐bound species were notably more prominent in the A‐series (synthesized at 25°C) than in the T‐series (synthesized at 80°C), indicating that elevated synthesis temperatures promote a more effective removal or decomposition of organic residues prior to calcination.

The presence of pronounced Zn─O bands, coupled with the absence or minimal intensity of organic vibrational modes, provides compelling evidence for the formation of phase‐pure ZnO NPs with negligible residual organic content.

### 3.2 XRD Analysis

**FIGURE 2:**
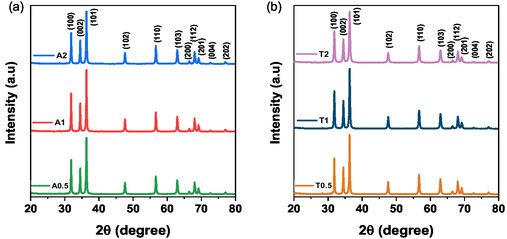
XRD patterns of ZnO NPs synthesized at (a) 25°C, A‐series, (b) at 80°C, T‐series.

The crystal structure, phase purity, and average crystallite size of the synthesized ZnO NPs were analyzed using XRD spectroscopy. The representative XRD patterns are shown in (Figure 2). All the diffraction patterns confirmed the successful formation of phase‐pure hexagonal wurtzite ZnO, consistent with JCPDS Card No. 36–1451 [37].

Well‐defined diffraction peaks corresponding to characteristic crystallographic planes, such as (100), (002), (101), (102), (110), (103), and (112), were observed, with a notable absence of detectable impurity phases across all samples. The sharpness of these peaks generally indicates the good crystallinity of the synthesized materials. Variations in peak broadening were observed, from which the average crystallite size (*D*_mean_) was estimated using the Scherrer equation (Equation (7)) [21].

(7) $$D=\;\frac{K\times \lambda }{\beta \times \text{cos}\;\theta }$$

where *K* represents the shape factor, valued at 0.9, and *λ* denotes the X‐ray wavelength, specifically 1.5406 Å for Cu Kα. The variable *β* corresponds to the full width at half maximum (FWHM) measured in radians, and *θ* signifies the Bragg diffraction angle, which is applied to the three most intense reflections: (100), (002), and (101). Table 1 presents the specific peak positions (2*θ*), corresponding Miller indices (hkl), FWHM values, calculated interplanar spacings (*d*_(hkl)_), derived lattice parameters (a, c), and crystallite sizes (*D* from individual peaks and the average *D*_mean_).

**TABLE 1:** Structural parameters derived from the XRD analysis of the synthesized ZnO NPs (A0.5‐T2), including 2θ positions, Miller indices (hkl), FWHM, interplanar spacings (*d*_(hkl)_), lattice parameters (a, c), and crystallite sizes (*D*, *D*_mean_).

Samples	2θ, °	(hkl)	FWHM, °	***d***_**(hkl)**_	*a*, nm	*c*, nm	*D*, nm	*D*_mean_ **, nm**
A0.5	31.84	(100)	0.25	0.2807	0.3242	0.5194	29.7	28.6 ± 0.7
	34.50	(002)	0.26	0.2597			28.8	
	36.33	(101)	0.27	0.2470			27.2	
A1	31.84	(100)	0.23	0.2807	0.3241	0.5194	32.7	31.2 ± 0.9
	34.50	(002)	0.24	0.2597			31.3	
	36.33	(101)	0.25	0.2470			29.6	
A2	31.84	(100)	0.25	0.2807	0.3241	0.5194	29.9	28.4 ± 0.8
	34.50	(002)	0.26	0.2597			28.3	
	36.33	(101)	0.27	0.2470			27.2	
T0.5	31.83	(100)	0.25	0.2809	0.3243	0.5196	30.0	28.5 ± 0.9
	34.48	(002)	0.26	0.2598			28.6	
	36.31	(101)	0.28	0.2471			26.8	
T1	31.85	(100)	0.28	0.2807	0.3241	0.5193	26.6	25.3 ± 0.8
	34.50	(002)	0.29	0.2596			25.5	
	36.34	(101)	0.31	0.2470			24.0	
T2	31.85	(100)	0.33	0.2807	0.3241	0.5194	22.7	21.6 ± 0.7
	34.50	(002)	0.35	0.2597			21.3	
	36.33	(101)	0.36	0.2470			20.4	

The average crystallite size (*D*_mean_) for the A‐series exhibited a nonmonotonic response to the precursor concentration. Specifically, *D*_mean_ increased from 28.6 ± 0.7 nm (A0.5) to 31.2 ± 0.9 nm (A1) before decreasing to 28.4 ± 0.8 nm (A2). This trend suggests a transition from a growth‐favored regime to a nucleation‐dominated process at higher supersaturation [38]. This behavior is consistent with solution‐phase synthesis, where the balance between nucleation and growth kinetics is concentration‐dependent. In contrast, at elevated temperature (T‐series), a systematic decrease in *D*_mean_ was observed with increasing precursor concentration. Specifically, *D*_mean_ was reduced from 28.5 ± 0.9 nm (T0.5) to 25.3 ± 0.8 nm (T1), and then to 21.6 ± 0.7 nm (T2). This consistent reduction at 80°C can be attributed to thermal activation, which lowers the nucleation energy barrier and promotes nucleation‐dominated kinetics across the entire concentration range examined [39]. Consequently, a higher number of nuclei are formed, leading to a distribution of the available solute among a larger population of particles, thus yielding smaller average crystallite sizes.

In summary, the combined influence of the precursor concentration and temperature revealed a distinct interdependence. While temperature exerted a minimal effect on the crystallite size (~28 nm) at low concentration (0.5 M), its impact became increasingly pronounced at higher concentrations (1 and 2 M). At these levels, the synergy between the elevated temperature and precursor concentration appears to accelerate the nucleation kinetics, yielding significantly smaller crystallites. Furthermore, the lattice parameters a and c remained stable and closely matched the standard values for hexagonal wurtzite ZnO [21], confirming the preservation of the phase purity and structural integrity.

### 3.3 Optical Properties and Bandgap Analysis

The optical absorption characteristics of the biosynthesized ZnO NPs were examined using UV–vis spectroscopy, and the resulting absorbance spectra and corresponding Tauc plots are depicted in Figures S1 (ESI) and 3, respectively.

**FIGURE 3:**
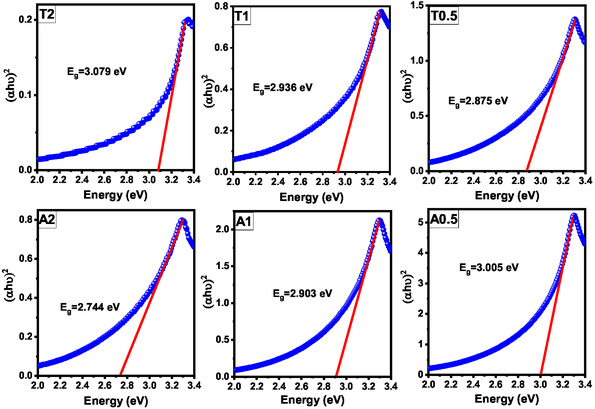
Tauc plots (αhν)^2^ versus photon energy (hν) for the estimation of the optical bandgap (*E*_g_) of the ZnO samples.

All the samples exhibited strong ultraviolet absorption with distinct excitonic features in the 372–378 nm range, indicating high‐quality crystalline wurtzite ZnO [40]. As illustrated in Figure S1, a notable redshift in the absorption edge was observed, particularly for sample A2, which aligns with the narrowed bandgap of 2.744 eV derived from the Tauc plots (Figure 3). These shifts underscore the influence of the synthesis parameters on the optical properties, likely due to variations in morphology, crystallite size, and defect density, which collectively modify the electronic structure [41].

The optical bandgap energies (*E*_g_) were determined from the Tauc plots (Figure 3) by extrapolating the linear segment of (αhν)^2^ versus photon energy (hν) to the x‐intercept. A clear dependence of *E*_g_ on both the precursor concentration and synthesis conditions was observed. For the A‐series (synthesized at 25°C), an increase in the precursor concentration resulted in a systematic narrowing of the bandgap, with *E*_g_ decreasing from 3.005 to 2.744 eV (A0.5 to A2, respectively). Conversely, the T‐series (80°C) exhibited the opposite trend, where *E*_g_ increased from 2.875 eV (T0.5) to 3.079 eV (T2) as the precursor concentration increased. Although these *E*_g_ values are significantly lower than those of bulk ZnO (~3.37 eV), such deviations are commonly reported for biogenic nanocrystalline ZnO samples and are attributed to a combination of high surface‐to‐volume ratios and structural defects [42, 43]. This redshift is consistent with several recent studies documenting comparable bandgap narrowing in green‐synthesized ZnO, such as Maind et al. (2.74 eV) [44], Al‐Harbi et al. (2.67 eV) [45], and Kolotygina et al. (2.85 eV) [46]. The further clarification of the narrowing of the absorption edge is explained by the introduction of specific lattice defects and surface states in the electronic structure. As further elucidated by EDS analysis in Section 3.5.3, the oxygen‐rich stoichiometry of our samples indicates that these defect states predominantly comprise oxygen interstitials (*O*_*i*_) and zinc vacancies (*V*_*Zn*_). These defects create sub‐gap energy levels that effectively reduce the energy required for electronic transitions. Furthermore, the presence of residual carboxylate groups (COO^‐^), identified through FTIR analysis in Section 3.1, contributes to this narrowing by creating localized states near the band edges. The pronounced narrowing observed in the A‐series indicates that ambient‐temperature synthesis promotes a higher density of these structural defects and preserves the bio‐organic interfaces. Conversely, the upward trend in *E*_g_ for the T‐series implies that higher synthesis temperatures (80°C) facilitate structural rearrangement and a relative reduction in defect densities, shifting the electronic properties back toward bulk‐like characteristics as the concentration increases. This divergent behavior underscores the precise modulation of the electronic properties of PPE‐derived ZnO by the thermal synthesis environment.

### 3.4 Textural Properties and Surface Area Analysis (BET/BJH)

**FIGURE 4:**
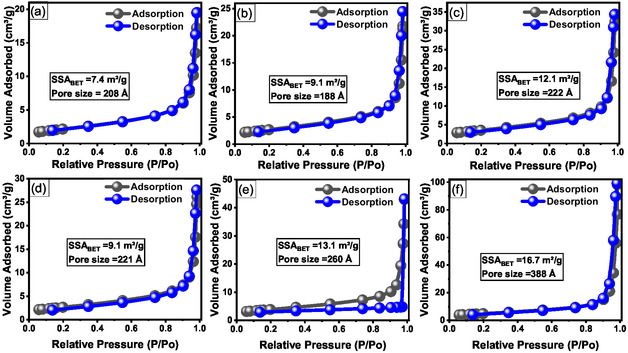
N_2_ adsorption–desorption isotherms of biosynthesized ZnO NPs: (a–c) A‐series (ambient) and (d–f) T‐series (80°C).

The textural properties of the biosynthesized ZnO NPs were assessed using N_2_ adsorption–desorption isotherms (Figure 4). All samples exhibited Type IV isotherms with H3 hysteresis loops, as per the IUPAC classification, indicative of mesoporous structures (pore widths 2–50 nm) [47]. The presence of a distinct H3 loop, typically associated with slit‐like pores formed by the aggregation of nonspherical particles, suggests that the observed porosity is predominantly interparticle in nature rather than arising from intrinsic mesoporosity within the ZnO crystallites. The BET specific surface area (SSA_BET_) increased systematically with the precursor concentration for both series. The values ranged from 7.4 m^2^/g (A0.5) to 12.1 m^2^/g (A2) for the A‐series and showed a more pronounced increase for the T‐series, reaching a maximum of 16.7 m^2^/g (T2). This latter value is in excellent agreement with our previous findings for starch‐biosynthesized ZnO [21]. This increasing trend in SSA_BET_ correlates inversely with the XRD‐derived crystallite sizes (21.6 to 31.2 nm), confirming the physical principle that smaller primary particles yield higher surface‐to‐volume ratios [48]. Notably, the T‐series samples exhibited SSA_BET_ values up to 38% higher than their A‐series counterparts at equivalent concentrations (specifically at 2 M).

The average interparticle pore diameters (*d*_p_) were determined using the BJH method applied to the desorption branch of the isotherms. The A‐series exhibited a nonmonotonic variation in *d*_p_ (20.8, 18.8, and 22.2 nm for A0.5, A1, and A2, respectively), likely reflecting the stochastic nature of particle aggregation at ambient temperature. In contrast, the T‐series demonstrated a systematic increase in *d*_p_ with precursor concentration, extending from 22.1 nm (T0.5) to 38.8 nm (T2). This finding suggests that under a nucleation‐dominated regime at 80°C, the assembly of smaller primary particles favors the formation of more open secondary structures with larger interparticle voids compared to closer packing typically achieved by larger crystallites. Specifically, for sample T2, the measured interparticle pore diameter (38.8 nm) significantly exceeded the primary crystallite size (21.6 nm). This is physically consistent with a highly open hierarchical architecture in which fine primary NPs are loosely branched, creating large interstitial gaps, as evidenced by FESEM and STEM observations. In summary, synthesis at 80°C with elevated precursor concentrations promoted the formation of smaller crystallites that assembled into architectures with superior surface areas and larger interparticle pores, whereas ambient conditions favored more compact arrangements with lower porosity.

### 3.5 Morphological and Compositional Characterization

The morphology, nanostructure, aggregation, texture, and elemental composition of the synthesized ZnO NPs were characterized using FESEM, STEM, and EDS.

#### 3.5.1 FESEM Analysis

**FIGURE 5:**
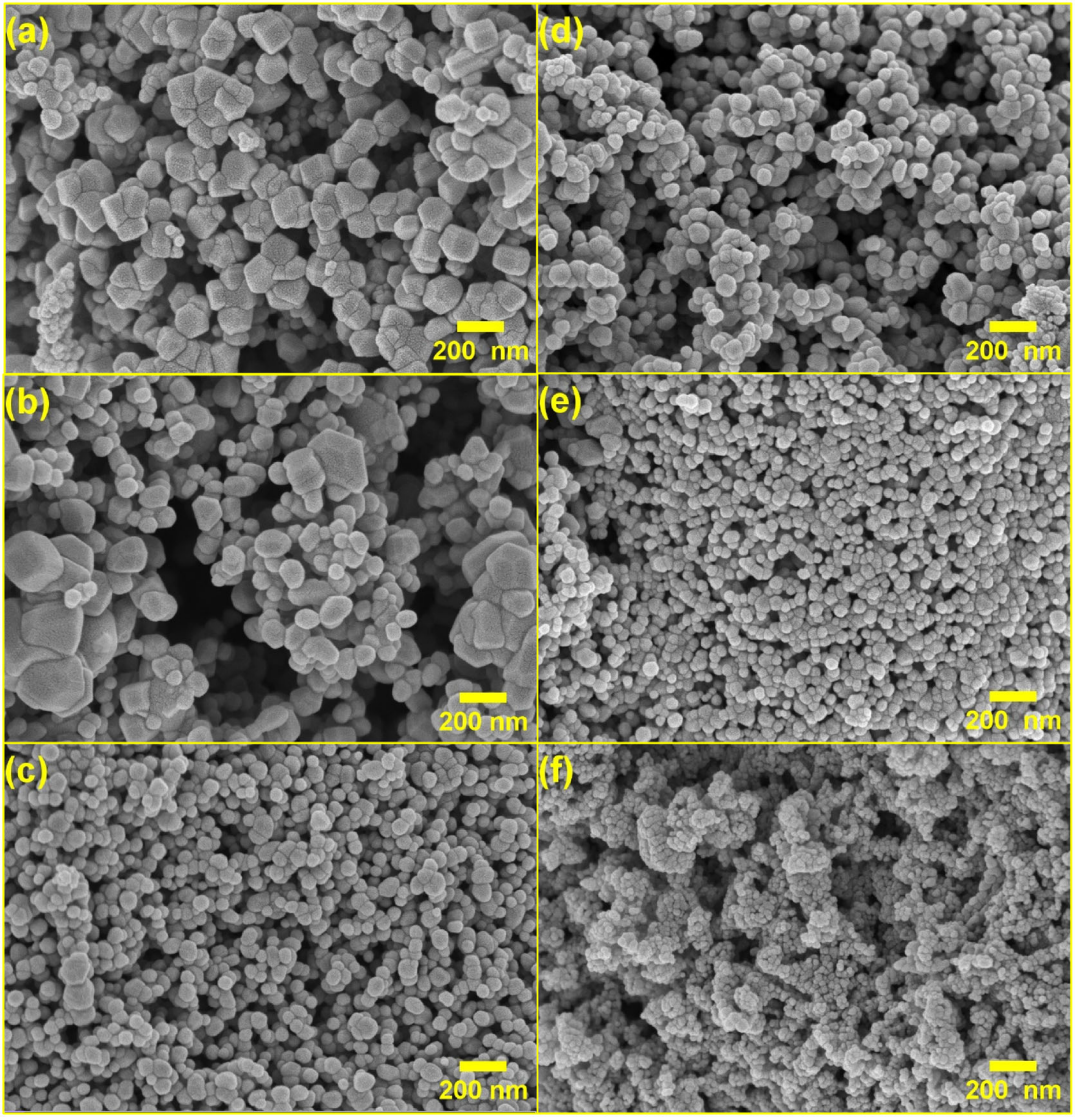
FESEM images of synthesized ZnO NPs: (a) A0.5, (b) A1, (c) A2, (d) T0.5, (e) T1, (f) T2.

FESEM imaging (Figure 5) elucidated distinct morphological transformations influenced by both the synthesis temperature and precursor concentration. The A‐series samples demonstrated a progressive transition in surface architecture. At the lowest concentration (A0.5, Figure 5a), the material consisted of relatively large, well‐defined quasi‐spherical and polyhedral particles forming loose agglomerates. Increasing the concentration to 1 M (A1, Figure 5b) resulted in a more textured morphology characterized by denser clusters, providing a moderately porous framework. At the highest concentration (A2, Figure 5c), the apparent particle size became markedly finer, forming an extensively interconnected porous network. Although the XRD results (Table 1) indicate a larger crystallite size for A1 (31.2 nm) than for A2 (28.4 nm), the FESEM images suggest that the visual refinement in A2 originates from a hierarchical assembly with crystallites organizing into a more interconnected and agglomerated framework. This specific arrangement results in the most developed porosity within the ambient series. In contrast, the T‐series exhibited a more systematic morphological evolution. At 0.5 M (T0.5, Figure 5d), primary particles were relatively small, and their loose packing generated significant interparticle voids. Increasing the concentration to 1 M (T1, Figure 5e) led to a more intricate porous framework, ultimately resulting in the exceptionally fine NPs observed at 2 M (T2, Figure 5f). This configuration demonstrates that the synergy between elevated temperature and high precursor concentration yields a highly open hierarchical architecture, accounting for the significantly larger interparticle pore diameters (388 Å) and the highest surface area (16.7 m^2^/g). Overall, the FESEM analysis confirms that synthesis conditions can be synergistically employed to tune ZnO morphology, offering diverse structural characteristics tailored to specific functional requirements.

#### 3.5.2 STEM Analysis

**FIGURE 6:**
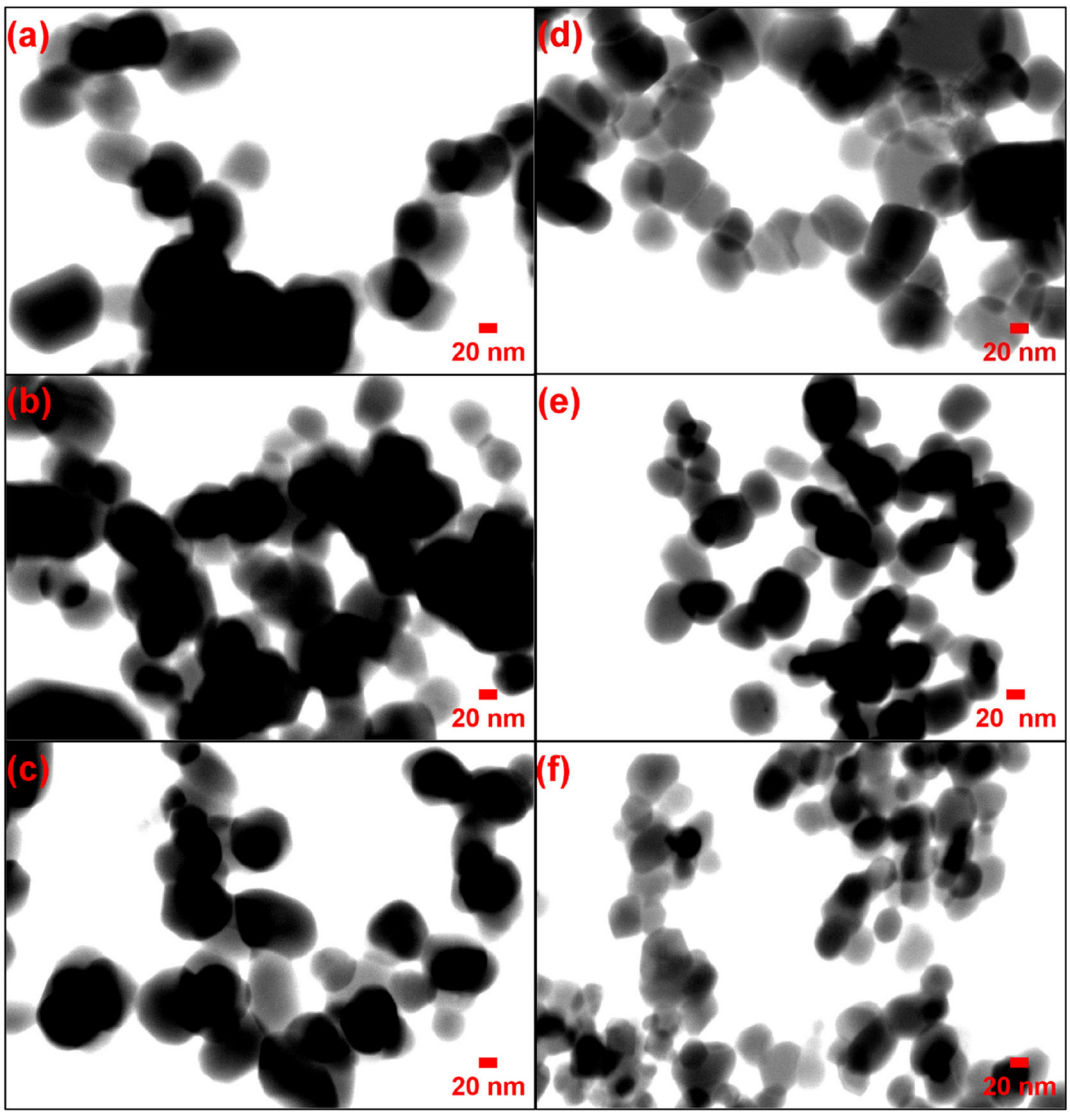
STEM images of synthesized ZnO NPs: (a) A0.5, (b) A1, (c) A2, (d) T0.5, (e) T1, (f) T2.

STEM was employed to elucidate the morphology and crystallinity at high resolution, thereby corroborating the primary particle dimensions (Figure 6).

In the A‐series, sample A0.5 (Figure 6a) exhibited discrete primary NPs with dimensions in good concordance with the crystallite size derived from XRD analysis (28.6 nm). As the precursor concentration increased, samples A1 (Figure 6b) and A2 (Figure 6c) exhibited more pronounced agglomeration. Notably, although A2 appeared visually more refined and compactly clustered than A0.5, STEM analysis indicated that this effect stemmed primarily from a denser spatial arrangement and particle packing rather than a substantial reduction in primary crystallite size, as A2 maintained a crystallite dimension (28.4 nm) nearly identical to that of A0.5. STEM imaging provided further insight into the systematic structural evolution of the T‐series. Sample T0.5 (Figure 6d) comprised well‐defined primary NPs, consistent with the XRD results (28.5 nm). With increasing precursor concentration, samples T1 (Figure 6e) and T2 (Figure 6f) exhibited a clear and progressive reduction in primary particle size, in strong agreement with the XRD data (25.3 and 21.6 nm, respectively). Among these, T2 displayed the finest primary NPs, confirming that an elevated synthesis temperature combined with a high precursor flux promotes the formation of smaller primary crystallites that assemble into compact secondary aggregates. Furthermore, the electron‐dense appearance of the NPs observed in the STEM images is indicative of high material density and structural integrity [49].

#### 3.5.3 Elemental Composition (EDS Analysis)

Elemental analysis via EDS was conducted to verify the chemical composition of the synthesized ZnO NPs. Representative EDS spectra (Figure S2) and quantitative analysis confirmed that zinc (Zn) and oxygen (O) were the sole primary constituents, indicating the high elemental purity of all samples. The Zn:O atomic ratios revealed a notable excess of oxygen relative to the ideal 1:1 stoichiometry of bulk ZnO. This oxygen enrichment can be attributed to the high surface‐to‐volume ratio of the NPs, which facilitates the adsorption of atmospheric species. Given the typical sampling depth of EDS relative to the nanoparticle size range (21–31 nm), the analysis predominantly probes surface‐enriched regions, including adsorbed oxygen, chemisorbed water, and trace residual organic species [50]. Additionally, the oxygen‐rich composition likely arises from intrinsic lattice defects, such as oxygen interstitials (*O*_*i*_) and zinc vacancies (*V*_*Zn*_), which typically emerge during the synthesis and calcination of ZnO NPs. Consequently, the EDS results confirm the successful formation of ZnO, suggesting a chemically reactive surface environment modulated by the synthesis conditions.

### 3.6 ZP Analysis

**FIGURE 7:**
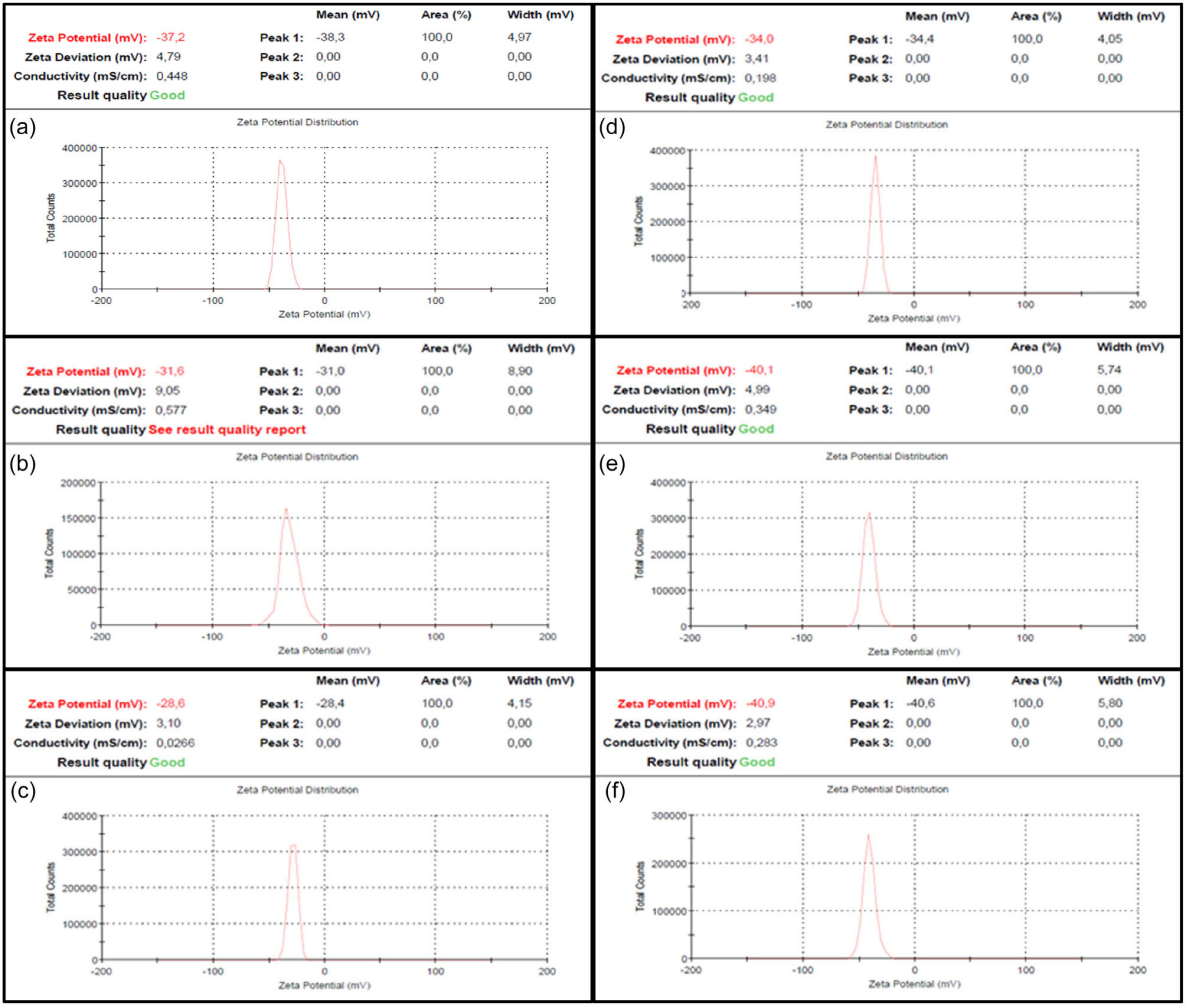
ZP distributions of ZnO NPs: (a) A0.5, (b) A1, (c) A2, (d) T0.5, (e) T1, (f) T2.

ZP measurements were conducted to evaluate the colloidal stability and surface charge characteristics of the biosynthesized ZnO NPs (Figure 7). All samples exhibited a net negative surface charge in aqueous media, with ZP values ranging from –28.6 mV (A2) to 40.9 mV (T2). These magnitudes indicate good‐to‐excellent stability, as electrostatic repulsion effectively counters van der Waals attractive forces to prevent significant aggregation. For the A‐series, the negative ZP is primarily extrinsic in nature, driven by residual biomolecules from the PPE. FTIR analysis of these samples revealed prominent absorption bands (1375–1521 cm^−1^) characteristic of the carboxylate groups (COO^−^). The deprotonation of these surface‐bound organic acids (R‐COOH → R‐COO^−^ + H^+^) is proposed as the primary source of the observed negative charge in the ambient series [51]. In stark contrast, the T‐series samples synthesized at 80°C did not exhibit carboxylate signatures in their FTIR spectra, suggesting the thermal decomposition or effective removal of these organic capping agents. Surprisingly, these “cleaner” T‐series NPs demonstrated significantly more negative ZP values (reaching –40.9 mV for T2) than their A‐series counterparts did. This counterintuitive finding indicates a transition to a dominant intrinsic mechanism for surface charge generation in the T‐series. This behavior is consistent with an oxygen‐rich or zinc‐deficient surface, as supported by the EDS analysis. The literature suggests that defect‐rich ZnO surfaces, particularly those with a high density of oxygen interstitials (*Oi*) or zinc vacancies (*V*_Zn_), can lead to a higher density of deprotonation sites (Zn‐OH → Zn‐O^−^ + H^+^) in aqueous media [52]. Consequently, the removal of the organic masking layer at 80°C likely exposes a highly reactive defect‐terminated surface that facilitates more efficient hydroxyl deprotonation, resulting in the superior electrostatic stabilization observed for sample T2. Overall, this systematic variation in the ZP highlights the complex interplay between adsorbed biomolecules and the intrinsic surface properties of ZnO. Understanding this mechanism switch in surface chemistry is paramount for optimizing material performance in charge‐sensitive applications such as photocatalysis and supercapacitance.

### 3.7 Interdependence of Synthesis Conditions on ZnO NP Properties

The cross‐sectional analysis of the physicochemical properties demonstrated that the synthesis temperature and precursor concentration synergistically influenced the nucleation and growth kinetics, thereby determining the distinct structural and electronic characteristics (Table 2 and Figure S3).

**TABLE 2:** Key physicochemical properties of ZnO NPs as a function of synthesis conditions.

Sample name	A0.5	A1	A2	T0.5	T1	T2
Precursor concentration, M	0.5	1	2	0.5	1	2
*E*_g_, eV	3.005	2.903	2.744	2.875	2.936	3.079
SSA_BET_, m^2^/g	7.4	9.1	12.1	9.1	13.1	16.7
Pore size, Å	208	188	222	221	270	388
Cristallite size, nm	28.6	31.2	28.4	28.5	25.3	21.6
ZP, mV	−37.2	−31.6	−28.6	−34.4	−40.1	−40.6

At 25°C in the A‐series, an increase in precursor concentration led to a systematic reduction in the optical bandgap (*E*_g_), achieving a minimum value of 2.744 eV for sample A2. This optimized band structure is expected to enhance photon harvesting compared to other variants. Although the T‐series (80°C) exhibited the highest specific surface area (reaching 16.7 m^2^/g for T2), its architecture remained fragmented and discrete, as evidenced by FESEM and STEM analyses. In contrast, sample A2 demonstrated a highly interconnected hierarchical architecture. The persistence of biogenic carboxylate groups on the surface of A2 (confirmed by FTIR, 1375–1521 cm^−1^) acting as “molecular bridges” suggests favorable surface chemistry for interfacial processes. Furthermore, EDS analysis confirms an oxygen‐rich stoichiometry for A2, indicating the predominance of oxygen interstitials (*O*_*i*_) and zinc vacancies (*V*_*Zn*_). In summary, the synergy between a narrowed *E*_g_ (2.744 eV), robust structural interconnectivity, and a biogenic interface rich in reactive defects positions sample A2 as the most promising candidate for functional applications. The validation of these structural advantages in photocatalytic and electrochemical performance will be discussed in the following sections.

### 3.8 Proposed Formation Mechanism: Influence of Temperature and Precursor Concentration

The biosynthesis of ZnO NPs commences through the interaction of phytochemicals present in the PPE, predominantly polyphenols such as ellagic acid and punicalagins [53], with Zn^2+^ ions. These biomolecules act as chelating and capping agents, promoting the formation of zinc–polyphenolate complexes [54, 55, 56]. At 80°C (T‐series), the elevated thermal energy enhances diffusion, facilitates more efficient ligand coordination, and ensures controlled complex hydrolysis, leading to the homogeneous nucleation of amorphous zinc hydroxide/oxyhydroxide intermediates [56]. The elevated nucleation rate, further amplified by the increased precursor concentration, results in a higher density of nuclei, thereby yielding smaller crystallite sizes that decrease from 28.5 to 21.6 nm for T0.5→T2 (Table 1). The residual phytochemical species adsorbed on these nascent crystallites effectively restricted grain growth, contributing to the observed increase in surface area and porosity (Section 3.4). In contrast, at 25°C (A‐series), reduced thermal activation leads to slower diffusion, less efficient Zn^2+^–ligand coordination, and more heterogeneous nucleation events [55]. This resulted in less controlled particle formation and increased variability in crystallite dimensions, as evidenced by the measurements of 28.6, 31.2, and 28.4 nm for A0.5, A1, and A2, respectively (Table 1), and higher levels of aggregation due to weaker initial stabilization (Figures 5 and 6). Following the initial synthesis, the products were calcined at 600°C to fully decompose the zinc–phytochemical complexes and mineralize the remaining organic ligands. This transformation was evidenced by the significant attenuation or disappearance of the characteristic C─O and C─H vibrational bands in the FTIR spectra post‐calcination, with only minor bands near 1520 and 1375 cm^−1^ persisting owing to residual surface‐bound carboxylate (COO^−^) species derived from the phytochemical precursors. The calcination step induced the crystallization of the amorphous intermediates into the thermodynamically stable hexagonal wurtzite ZnO phase, as confirmed by XRD patterns consistent with JCPDS 36–1451 (Figure 2). Notably, EDS analysis revealed an oxygen‐rich surface, attributed to the presence of multiple oxygenated species adsorbed on the nanoparticle surface, including chemisorbed O_2_, hydroxyl groups, carbonates, and residual carboxylates, reflecting the high surface reactivity typical of nanoscale metal oxides [55]. Overall, the initial synthesis temperature and precursor concentration are decisive determinants of the final nanoparticle dimensions, textural characteristics, and, significantly, their defect profiles (including oxygen vacancies [57]) and surface chemistry.

### 3.9 Photocatalytic Performance Evaluation

#### 3.9.1 Degradation of MB Using Synthesized ZnO NPs

**FIGURE 8:**
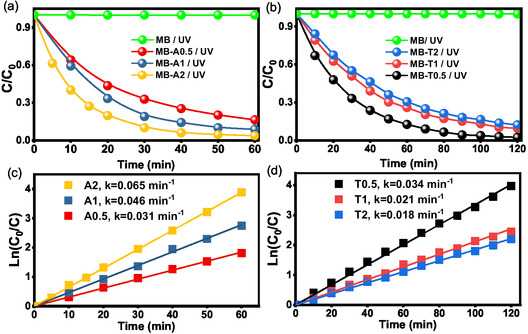
Photocatalytic degradation of MB under UV light. (a) *C*/*C*_0_ versus time for ambient temperature synthesized ZnO (A‐series) compared with photolysis control, (b) *C*/*C*_0_ versus time for 80°C synthesized ZnO (T‐series) compared with photolysis control, (c and d) Pseudo‐first‐order kinetic plots (ln(*C*_0_/*C* ) vs. time) for A‐ and T‐series, respectively.

The photocatalytic degradation of methylene blue (MB, 10 ppm) was systematically investigated using ZnO NPs synthesized at various temperatures and precursor concentrations. Control experiments involving direct UV photolysis without a catalyst resulted in negligible degradation (<4% over 120 min), underscoring the critical role of ZnO NPs in facilitating efficient photocatalytic activity. Temporal evolution of MB absorbance at 652 nm (Figure S4) shows markedly faster decay for A‐series (Figure S4a–c) than T‐series (Figure S4d–f) NPs. This behavior underscores the strong impact of synthesis conditions on the physicochemical properties of the NPs, particularly crystallinity, surface defect density, and accessible active surface area, which collectively govern the efficiency of reactive oxygen species (ROS) generation and, consequently, the photocatalytic degradation rate [58]. The reaction kinetics (Figure 8 and Table 3) further support the trends identified in the UV–vis absorption evolution, reinforcing the observed differences in the degradation efficiency among the samples.

**TABLE 3:** Pseudo‐first‐order rate constants for MB photocatalytic degradation by synthesized ZnO NPs.

Sample name	A0.5	A1	A2	T0.5	T1	T2
*k*_app_, min^−1^	0.031	0.046	0.065	0.034	0.021	0.018

For the A‐series NPs, the photocatalytic activity increased systematically with increasing precursor concentrations. After 60 min of UV irradiation, the MB degradation efficiency followed the order A2 > A1 > A0.5 (Figure 8a). Correspondingly, the pseudo‐first‐order rate constants (*k*_app_) were determined to be 0.065, 0.046, and 0.031 min^−1^ for A2, A1, and A0.5, respectively (Figure 8c and Table 3). Notably, A2 exhibited the highest degradation efficiency of 97%, compared with 92% for A1 and 90% for A0.5. These results indicate that the photocatalytic degradation of MB is strongly governed by the intrinsic material properties, including crystallite size, specific surface area, porosity, and bandgap energy, all of which collectively control the light‐harvesting efficiency and kinetics of the generation, separation, transport, and recombination of photogenerated charge carriers.

In this context, the superior photocatalytic performance of A2 can be attributed to the synergistic interplay between its optimized electronic structure and hierarchical morphology. Specifically, A2 possesses the narrowest bandgap energy (*E*_g_ = 2.744 eV), which significantly enhances photon harvesting and maximizes the generation of the charge carriers. Simultaneously, its smallest crystallite size (28.4 nm) results in the highest specific surface area (12.1 m^2^ g^−1^) among the A‐series samples, providing a greater density of accessible active sites and promoting the efficient separation and migration of electron–hole pairs. Moreover, the interconnected hierarchical architecture of A2, identified as an “electronic highway” via FESEM and STEM, facilitates the rapid migration of electron–hole pairs to the surface while minimizing the internal resistive barriers, as confirmed by its near‐zero *R*_p_ in the EIS analysis (Section 3.10.3). These combined structural and electronic advantages strengthen the interaction between the photocatalyst surface and MB molecules, suppress charge recombination, and accelerate the photocatalytic degradation kinetics. This synergistic interplay among the surface charge, adsorption–desorption dynamics, and charge carrier kinetics is characteristic of heterogeneous photocatalysis [59].

In contrast to the A‐series, the T‐series NPs exhibited a systematic inverse correlation between the precursor concentration and photocatalytic activity (Figure 8b). After 120 min of UV irradiation, the degradation kinetics followed the order T0.5 > T1 > T2, with *k*_app_ values of 0.034, 0.021, and 0.018 min^−1^, respectively (Figure 8d and Table 3). Consistently, T0.5 exhibited the highest degradation efficiency (97%), outperforming T1 (91%) and T2 (90%). These results indicate that increasing the precursor concentration at 80°C adversely affects the photocatalytic performance, with T0.5 exhibiting optimal activity. Notably, a “Connectivity versus Surface Area” paradox was observed: despite T2 presenting the highest specific surface area (16.7 m^2^g^−1^) and the smallest crystallite size (21.6 nm) within the T‐series, it exhibited the lowest activity. The superior activity was achieved by T0.5, which possessed the lowest bandgap energy (2.875 eV), the largest crystallite size (28.5 nm), and the lowest SSA_BET_ (9.1 m^2^ g^−1^). This apparent discrepancy suggests that the photocatalytic efficiency is primarily dictated by the electronic structure and connectivity rather than textural parameters alone. In particular, at higher precursor concentrations at 80°C (T2), the high thermal flux likely promotes excessive particle aggregation and an increase in deep‐level surface defects, which act as effective nonradiative recombination centers (charge traps). This “defect‐trapping” effect is further corroborated by T2's highly negative ZP (–40.6 mV) and colossally high polarization resistance (*R*_p_ = 3069.64 Ω), which collectively “choke” charge carrier migration and significantly diminish the photocatalytic efficiency [60]. In conclusion, synthesis at ambient temperature was enhanced by higher precursor concentrations, resulting in catalysts with superior ^•^OH‐mediated activities. In contrast, synthesis at elevated temperatures requires lower precursor concentrations to avoid detrimental structural or electronic effects that could impair performance.

#### 3.9.2 Radical Scavenging and Mechanistic Insight

**FIGURE 9:**
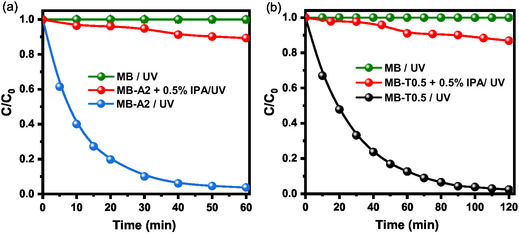
Photocatalytic degradation of MB under UV light. (a) *C*/*C*_0_ versus time for A2 sample compared with photolysis control and *A2 + 0.5% IPA* scavenger, (b) *C*/*C*_0_ versus time for T0.5 sample compared with *T0.5 + 0.5% IPA* scavenger.

To elucidate the primary oxidative species involved in this process, radical‐scavenging experiments were conducted using A2 and T0.5 samples. The addition of isopropyl alcohol (IPA, 0.5% v/v) as a ^•^OH quencher substantially inhibited the degradation rate, with suppression levels reaching 90% for A2 (Figure 9a) and 87.4% for T0.5 (Figure 9b). These findings confirm that hydroxyl radicals (^•^OH) are the predominant oxidative species responsible for MB degradation in both systems. This degradation pathway is generally understood to proceed via N‐demethylation, hydroxylation, and ring‐opening, potentially leading to mineralization [61]. Despite these inhibitory effects, a residual degradation efficiency of 10%–12.6% was still observed, indicating the contribution of secondary pathways, such as direct hole (h^+^) oxidation or alternative reactive radicals, such as superoxide anions ($O_{2}^{\cdot -}$).

**SCHEME 2:**
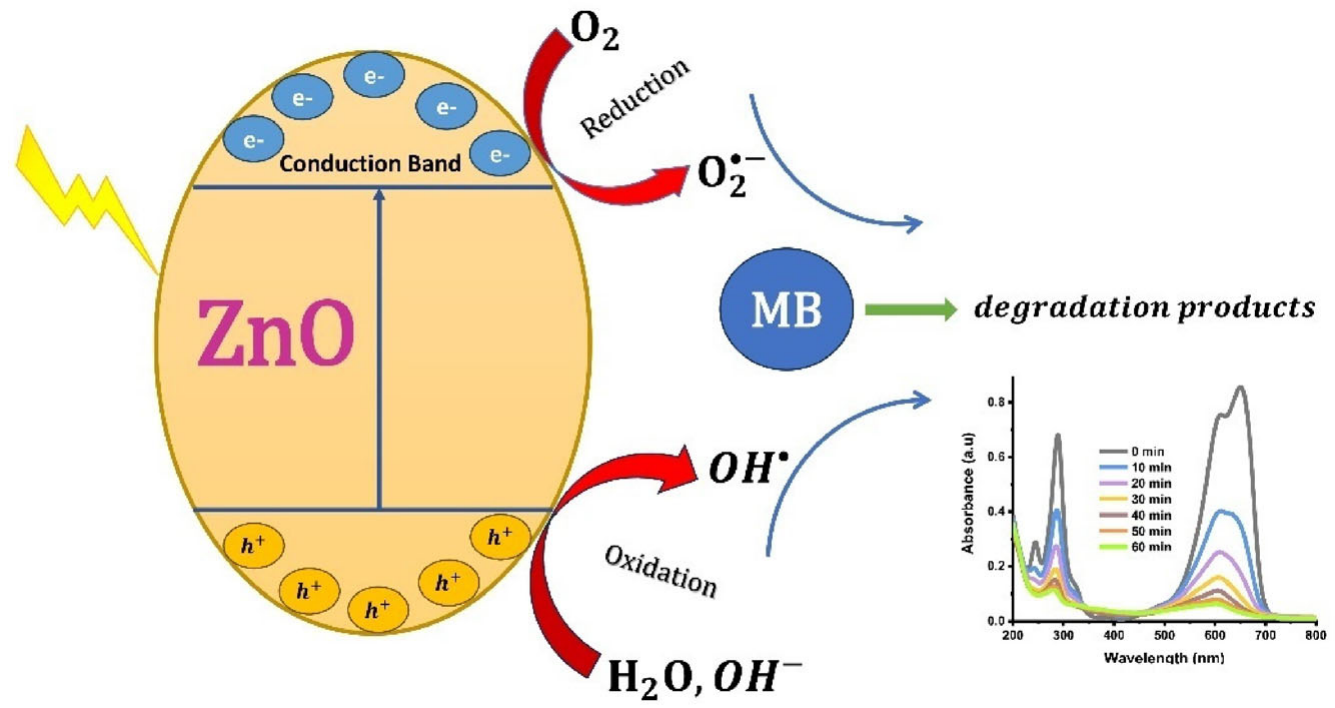
Proposed photocatalytic mechanism for MB degradation over biogenic ZnO NPs.

Based on this, a detailed photocatalytic mechanism is proposed, as shown in Scheme 2. Upon UV irradiation, ZnO NPs absorb photons with energy equal to or greater than their bandgap (hv ≥ *E*_g_), promoting electrons to the conduction band and leaving behind holes in the valence band (e^‐^/_CB_ and h^+^/_VB_). The superior photocatalytic performance of A2 stems from the synergy between its interconnected architecture and its biogenic interface; FTIR analysis confirmed that A2 preserves residual carboxylate groups (COO^‐^), which act as “molecular bridges,” facilitating charge transfer to the adsorbed dye molecules [62]. The photogenerated holes (h^+^) react with surface‐adsorbed water or hydroxide ions to produce reactive hydroxyl radicals (^•^OH). Concurrently, electrons in the conduction band reduce the dissolved oxygen to produce superoxide radicals ($O_{2}^{\cdot -}$ **).** These ROS synergistically attack MB molecules, degrading them into smaller intermediate fragments or benign products. This high functional efficiency is further balanced by the high environmental safety of the material (LC_50_ = 4842 µg/mL), ensuring sustainable deployment for environmental remediation. The photocatalytic process proceeds through the following fundamental steps:

(8) $$\mathrm{ZnO}+h\text{ν}\;\to \;\;e_{\mathrm{CB}}^{-}+\;h_{\mathrm{VB}}^{+}$$

(9) $$h_{\mathrm{VB}}^{+}+H_{2}O\;\to \;+\;{\mathrm{HO}}^{•}+\;H^{+}$$

(10) $$h_{\mathrm{VB}}^{+}+{\mathrm{OH}}^{-}\;\to \;+\;{\mathrm{HO}}^{•}$$

(11) $$e_{\mathrm{CB}}^{-}+\;O_{2}\;\to \;O_{2}^{•-}$$

(12) $${2O}_{2}^{•-}+\;2H^{+}\;\to \;H_{2}O_{2}+O_{2}$$

(13) $$H_{2}O_{2}+\;e_{\mathrm{CB}}^{-}\;\to \;{\mathrm{HO}}^{•}+{\mathrm{OH}}^{-}$$

(14) $$\text{ROS}({\mathrm{HO}}^{•},\;O_{2}^{•-},\;h^{+})+\text{MB dye}\;\to \;\text{degradation products}$$

#### 3.9.3 Recyclability

**FIGURE 10:**
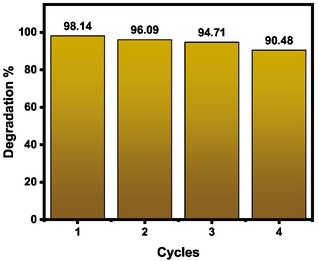
Recyclability of ZnO NPs (A2).

The photostability and reusability of the optimal ZnO catalyst (A2) were assessed over four consecutive cycles (Figure 10). Sample A2 was selected for these evaluations because it demonstrated the highest photocatalytic efficiency across both the A‐ and T‐series. The material exhibited exceptional durability, with the MB degradation efficiency undergoing only a minor attenuation, from 98% to 90.5% by the fourth cycle of use. This sustained high activity underscores the structural resilience and effective preservation of active surface sites even after repeated exposure to the reaction environment. These findings highlight the practical viability of the biosynthesized A2 catalyst, emphasizing its potential for long‐term environmental remediation and its capacity to enhance the economic and ecological feasibility of green‐mediated water treatment technologies**.**

### 3.10 Electrochemical Characterization of ZnO NPs

#### 3.10.1 CV

CV was employed to evaluate the electrochemical capacitive performance of the synthesized ZnO nanomaterials (A0.5, A1, A2, T0.5, T1, and T2). The CV profiles depicted in Figure S5 reveal distinct anodic and cathodic peaks for all ZnO electrodes, indicative of predominant pseudocapacitive charge storage mechanisms. This behavior, characterized by reversible Faradaic reactions involving ZnO, deviates significantly from the ideal rectangular shape of electric double‐layer capacitors; such a pseudocapacitive response is consistent with previous reports on green‐synthesized ZnO [21, 63, 64]. As illustrated in Figure S5, a systematic increase in the peak current and enclosed CV area with increasing scan rate (10–100 mV/s) was observed for all samples, signifying favorable capacitive behavior and a good rate capability.

**FIGURE 11:**
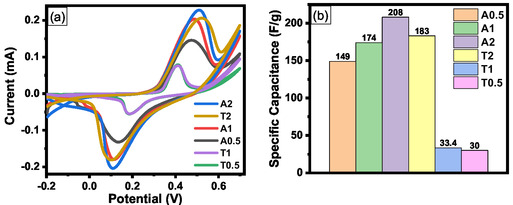
Comparison of electrochemical performances (a) CV plots (scan rate, 100 mV/s) (b) Specific capacitances at 1A/g.

Intra‐series comparisons demonstrated a consistent increase in the peak current with increasing precursor concentration (A0.5–A2 and T0.5–T2; Figure S5). This trend indicates that higher precursor concentrations facilitate the formation of a larger electroactive surface area with more accessible redox sites, thereby enhancing the interfacial ion transport kinetics and improving charge transfer efficiency. A discernible positive shift in the anodic peak potential (*Epa*) and a negative shift in the cathodic peak potential (*Epc*) with increasing scan rates were observed for all ZnO electrodes (Figure S5). This peak polarization typically indicates the presence of kinetic limitations in the electrochemical process, likely arising from the charge–transfer resistance or diffusion constraints within the electrode architecture [65, 66]. Among the samples, A2 exhibited the largest integrated CV area, followed by T2, A1, A0.5, T1, and T0.5 (Figure 11a). The performance advantage of A2, despite T2 having a higher nominal surface area, suggests that the robust interconnected hierarchical architecture of A2 provides superior electrical connectivity and more efficient charge transfer pathways than the T‐series. This sequence reflects the enhanced electrochemical performance, which is consistent with the highest specific capacitance derived from GCD measurements (Figure 11b).

**FIGURE 12:**
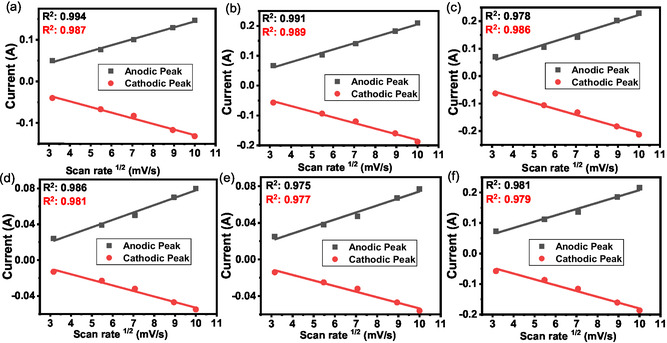
Linear relationship beLinear relationship between the peak currents (i_p_) and the square root of the scan rate (v^1/2^) for (a) A0.5, (b) A1, (c) A2, (d) T0.5, (e) T1, and (f) T2.

To elucidate the underlying charge‐storage mechanism, the relationship between the peak current (*i*_p_) and the square root of the scan rate (*v* ^*1/2*^) was analyzed (Figure 12). All samples demonstrated a strong linear correlation (*R* ^2^ > 0.9), indicating that the charge‐storage behavior is predominantly governed by ion diffusion, a characteristic of diffusion‐controlled electrochemical processes [62]. To further provide a quantitative assessment of the charge storage kinetics, a b‐value analysis was implemented using the power‐law relationship:

(15) $$i_{p}=a\times v^{b}$$

where (a) is a constant, and the exponent (b) is the slope of the log(*i*_p_) versus log(*v*) plot. Theoretically, a b‐value of 0.5 signifies a process that is entirely diffusion‐controlled (battery‐type behavior), whereas a b‐value of 1.0 denotes a surface‐controlled capacitive process [67]. For the synthesized ZnO series, the calculated b‐values ranged from 0.46 to 0.52, as shown in Figure S6. These values are close to 0.5, confirming that diffusion‐limited kinetics predominate in the pseudocapacitive response of these materials. This diffusion‐controlled mechanism is consistent with the hierarchical porous network observed by BET and FESEM analyses. Although the linear *i*_p_–*v* ^*1/2*^ relationship indicates that ion transport through the bulk of the electrode governs the process, the observed shifts in the peak potential (Figure S5) suggest that the overall rate capability is still affected by polarization effects. These encompass resistive barriers to ion migration within the electrode architecture and internal cell resistance (ohmic drop). As the measurements were conducted without iR correction to simulate practical operational conditions, these peak shifts offer a transparent depiction of the kinetic overpotentials and resistive contributions affecting the charge transfer efficiency and operational speed [65, 68].

#### 3.10.2 GCD

The electrochemical performance identified via CV was further validated through GCD measurements. Figure S7 shows the GCD profiles at various current densities (1, 2, 5, and 10 A/g) for all ZnO electrodes. The nonlinear quasi‐triangular configuration of these curves corroborates the significant contribution of pseudocapacitance to the charge storage mechanism, consistent with the CV results [69]. The specific capacitances (*C*_sp_) were determined from the discharge segments using Equation (3). At 1 A/g, the values were 208, 174, and 149 F/g for A2, A1, and A0.5, and 183, 33.4, and 30 F/g for T2, T1, and T0.5, respectively (Table 4). Notably, A2 achieved the highest capacitance (208 F/g), outperforming even T2 (183 F/g), despite its 38% lower specific surface area (12.1 vs. 16.7 m^2^/g)**.** This superiority of A2 is maintained across the practical operating range of 1–5 A/g confirms that structural interconnectivity outweighs nominal surface area in governing charge storage, a finding mechanistically validated by EIS (Section 3.10.3). The dramatic failure of T1 and T0.5 (30–33.4 F/g) underscores that a low precursor concentration at 80°C is detrimental, whereas a high concentration (T2) partially compensates through increased active site density without overcoming the fundamental limitation of a fragmented architecture.

**TABLE 4:** Specific capacitance (F/g) of ZnO electrodes at various current densities.

Current density, A/g	Specific capacitance, F/g					
	A2	A1	A0.5	T2	T1	T0.5
1	208	174	149	183	33.4	30
2	198	169	135	176	29.1	26
5	174	156	103	163	24.5	22
10	143	145	76	124	15	19

The observed capacitance attenuation with increasing current density (Table 4) is a characteristic feature of pseudocapacitive materials, primarily ascribed to the diffusion‐limited kinetics of electrolyte ions. At elevated charge/discharge rates, there is insufficient time for ions to penetrate the electrode bulk and complete Faradaic redox reactions, a process further exacerbated by increased internal resistance (ohmic drop) [66, 70]. Notably, samples A2 and T2 exhibited the highest *C*_sp_ values within their respective series at lower current densities (1–5 A/g), confirming that higher precursor concentrations enhance the density of electrochemically active sites. At 10 A/g, A1 marginally surpassed A2 (145 vs. 143 F/g), suggesting that at extreme current densities, ion diffusion limitations may temporarily offset the connectivity advantage; nevertheless, A2's overall dominance across the typical operating range (1–5 A/g) and its superior rate capability retention validate its status as the optimal electrode.

#### 3.10.3 EIS

**FIGURE 13:**
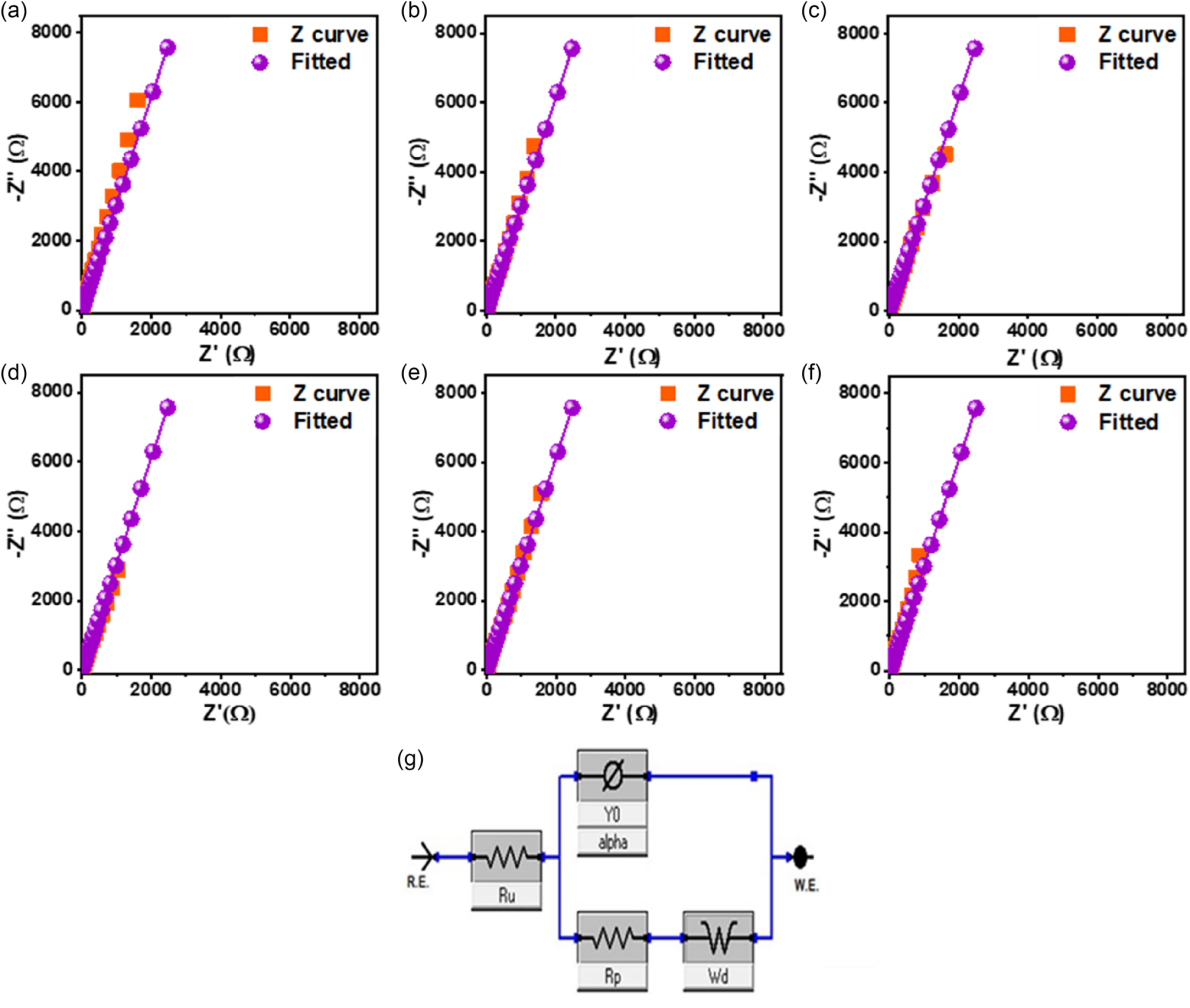
EIS analysis: (a–f) Nyquist plots of the biosynthesized ZnO electrodes (T0.5, T1, T2, A0.5, A1, and A2, respectively), and (g) the representative equivalent electrical circuit model employed for fitting the experimental data.

EIS was used to gain a comprehensive understanding of the charge–transfer kinetics and ion‐diffusion behavior of the synthesized ZnO NPs. The Nyquist plots (Figure 13) reveal qualitatively similar characteristics across all samples, featuring a depressed semicircle in the high‐frequency region, followed by a quasi‐linear segment in the low‐frequency region. The diameter of the semicircle corresponds to the polarization resistance (*R*_p_) at the electrode–electrolyte interface, whereas the low‐frequency linear portion represents the Warburg impedance (*W*_d_), which is indicative of ion diffusion within the electrode architecture [65, 71].

To quantitatively evaluate the electrochemical parameters, the EIS data were fitted using the equivalent electrical circuit (EEC) model, shown in Figure 13g. In this model, *R*_u_ represents the uncompensated solution resistance, *R*_p_ is the polarization (charge–transfer) resistance, *W*_d_ is the Warburg impedance, and *Y*_0_ (with the exponent α) is the constant‐phase element (CPE) used to account for the nonideal capacitive behavior and surface heterogeneity of the porous electrode [65, 71]. The extracted parameters are summarized in Table 5.

**TABLE 5:** EIS parameters extracted from the equivalent circuit fitting [*R*_u_(Q[*R*_p_ *W*_d_])].

Sample	***R***_**u**_ **, Ω**	***Y***_**0**_ **(S·s** ^**a**^ **)**	*α* (CPE exponent)	***W***_**d**_ **(S·s** ^**0.5**^ **)**	***R***_**p**_ **, Ω**
A0.5	0.7372	2.34 × 10^−^^3^	0.7716	2.84 × 10^−^^4^	115.43
A1	0.7832	2.12 × 10^−3^	0.8088	6.85 × 10^−12^	617.82
A2	0.8578	2.36 × 10^−3^	0.7873	1.26 × 10^−10^	0.0043
T0.5	0.7000	1.88 × 10^−3^	0.8124	6.65 × 10^−11^	991.29
T1	0.7506	2.31 × 10^−3^	0.8103	2.45 × 10^−11^	0.0001
T2	0.7312	2.40 × 10^−3^	0.7987	7.98 × 10^−11^	3069.64

A striking “Connectivity versus Surface area ” paradox emerges from the *R*_p_ analysis. Sample A2 exhibited an exceptional *R*_p_ of 0.0043 Ω, whereas T2 showed a colossal resistance of 3069.64 Ω, a difference of over six orders of magnitude. This disparity confirms that the fragmented morphology of T2, despite its 38% higher nominal surface area (16.7 vs. 12.1 m^2^/g), creates prohibitive grain‐boundary resistances that “choke” charge transport [65, 68], whereas A2's interconnected hierarchical architecture enables seamless electron migration. Notably, T1 displayed an anomalously low *R*_p_ (~0.0001 Ω) but exhibited negligible capacitance (33.4 F/g). This apparent contradiction demonstrates that ultralow charge–transfer resistance alone is insufficient without adequate electroactive site density. At a low precursor concentration (0.5 M), T1 lacked sufficient ZnO material to store the charge, regardless of the interfacial conductivity. Only A2 achieved the optimal synergy: a near‐ideal *R*_p_ (0.0043 Ω) combined with a high active‐site density (2 M precursor), resulting in a superior specific capacitance (208 F/g).

The CPE exponent (*α* ≈ 0.80) further reflects the surface heterogeneity and interparticle porosity characteristics of these biogenic ZnO electrodes. Residual polyphenolic and carboxylate groups in A2 (FTIR‐confirmed) act as “molecular bridges” that facilitate charge transfer, whereas A2's interconnected hierarchical architecture minimizes internal resistive barriers [71]. Crucially, while A0.5 and T1 show relatively low resistance, their specific capacitances (149 and 33.4 F/g, respectively) remain lower than that of A2 (208 F/g). This confirms that A2 achieves the optimal compromise between near‐ideal charge transfer kinetics and a high density of accessible electroactive sites provided by its robust interconnected framework. Collectively, these results underscore that connectivity and interfacial quality are more critical than the absolute surface area (SSA_BET_) for achieving high‐performance pseudocapacitance in green‐synthesized nanomaterials [72, 73].

#### 3.10.4 Energy and Power Density

**FIGURE 14:**
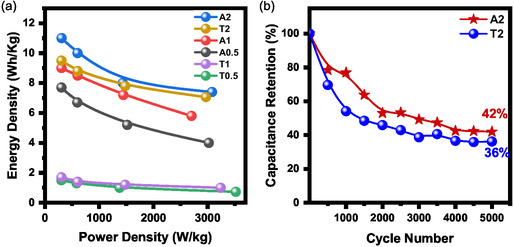
(a) Ragone plot illustrating the relationship between the energy density and power density for various ZnO electrodes. (b) Long‐term cycling stability and capacitance retention curves of the A2 and T2 electrodes over 5000 cycles at a high current density of 15 A g^−1^.

To assess the practical energy storage performance, energy density (E) and power density (P) were calculated from the GCD data using Equations (4) and (5) (Subsection 2.5.3). Figure 14a presents the Ragone plot, which illustrates the trade‐off between the energy and power density of the synthesized ZnO electrodes. Consistent with its superior specific capacitance, sample A2 exhibited the most prominent energy storage characteristics across the entire range examined. In particular, at a current density of 1 A/g, A2 achieved a maximum energy density of 11.0 Wh/kg at a power density of 308 W/kg. The Ragone plot reveals a characteristic inverse relationship between energy and power density, in which the energy density gradually decreases as the power density increases owing to internal resistance and diffusion constraints at higher discharge rates. The performance metrics of these ZnO nanostructures confirm their significant potential for application in supercapacitors. Moreover, future optimization of the electrode architecture and material nanostructuring could further improve these metrics, making them increasingly viable for high‐performance energy storage devices.

#### 3.10.5 Long‐Term Cycling Stability

The long‐term cycling stability of the A2 and T2 electrodes was evaluated over 5000 cycles using an accelerated stress test at an ultrahigh current density of 15 A g^−1^(Figure 14b). This rigorous condition was specifically selected to challenge the structural integrity and mechanical robustness of the biogenic ZnO framework under extreme power demand conditions. As illustrated in the capacitance retention curves, sample A2 maintained 42% of its initial capacitance, whereas sample T2 exhibited a lower retention of 36%. These values reflect the anticipated degradation of pure metal oxides in harsh alkaline media (2M KOH), primarily attributed to partial surface dissolution and mechanical strain arising from the intensive ion flux during ultrafast charge–discharge cycles. Notably, maintaining a 42% retention at such a formidable discharge rate is significant for pristine, carbon‐free biogenic materials, as it represents a “worst‐case scenario” evaluation. The superior resilience of A2 compared to T2 further validates that the interconnected hierarchical architecture synthesized at ambient temperature (25°C) provides an enhanced structural‐buffering effect. This robust network maintains electronic connectivity more effectively than the fragmented aggregates of the T‐series, which are more susceptible to mechanical crumbling under high‐velocity electrolyte fluxes. Furthermore, the stabilization trend observed after 3000 cycles suggests the attainment of a steady‐state electrochemical equilibrium. This high‐rate durability profile demonstrates that A2 is a promising candidate for applications requiring high‐power bursts, with substantial potential for further optimization through carbon‐based hybridization to mitigate surface dissolution and achieve commercial‐grade long‐term stability.

### 3.11 Comprehensive Structure‐Performance Correlation and Mechanistic Insight

Sample A2's superior performance stems from the synergy of its structural, electronic, and interfacial characteristics, rather than from a single parameter. To elucidate this, it is essential to critically correlate the roles of SSA_BET_, bandgap, crystallite size, defects, and residual biogenic species. A fundamental discovery of this study is the “connectivity versus surface area” paradox. Our results demonstrate that sample A2 consistently outperformed sample T2 in both applications, despite T2 possessing a 38% higher nominal surface area (16.7 m^2^/g) than A2 (12.1 m^2^/g). This indicates that morphological connectivity, rather than absolute surface area, dictates the “electronic highways” required for efficient charge transport. In sample A2, the interconnected hierarchical architecture synthesized at 25°C minimizes internal resistive barriers, as evidenced by its near‐zero polarization resistance (*R*_p_ = 0.0043 Ω), whereas the fragmented morphology of T2 leads to a colossally high resistance (*R*_p_ = 3069.64 Ω), effectively “choking” its functional performance. Second, the electronic band structure and defect engineering play crucial roles in dictating the reaction kinetics. The bandgap of A2 (2.744 eV) is the lowest among all the variants and is significantly narrower than that of T2 (3.079 eV). This narrowing shifts the absorption threshold toward the visible region, thereby enhancing the overall photon harvesting efficiency and facilitating the generation of electron–hole pairs. Based on EDS analysis, which revealed an oxygen‐rich surface, this narrowing is directly linked to intrinsic structural defects, such as oxygen interstitials (*O*_*i*_) or zinc vacancies (*V*_*Zn*_), which are generated during pomegranate‐mediated synthesis. Although the T‐series promotes smaller crystallites, the high‐temperature flux also introduces deep‐level defects that act as recombination centers, thereby quenching photocatalytic activity. In contrast, the ambient‐temperature synthesis of A2 preserves an optimized defect profile that provides beneficial active sites for both ROS generation and pseudocapacitive redox reactions.

Third, the biogenic interface functions as a “molecular bridge”. FTIR analysis revealed that the A‐series, particularly A2, retained residual carboxylate groups (COO^‐^) from the PPE, which would otherwise thermally decompose at higher temperatures. These residual species act as molecular bridges that facilitate interfacial charge transfer between the ZnO surface and electrolyte or pollutant molecules. This was supported by the stable negative ZP (–28.6 mV for A2), which ensures colloidal stability and enhances electrostatic attraction with the cationic MB dye.

In conclusion, A2 achieves the “golden compromise” required for bifunctionality: an interconnected architecture that outweighs the absolute surface area, a narrowed bandgap for enhanced optical response, and a bio‐functionalized surface that optimizes charge kinetics.

### 3.12 Ecotoxicity Assessment

The acute ecotoxicological profile of the biosynthesized ZnO NPs (sample A2), recognized as the most efficient catalyst in this study, was evaluated using a 24 h BSLA utilizing *Artemia salina* nauplii. A distinct concentration‐dependent toxicological response was observed, with mortality rates increasing progressively across the tested concentration range (125–4000 µg/mL) (Table 6).

**TABLE 6:** Effect of ZnO NPs (A2) concentration on Artemia salina nauplii mortality.

C, μg/mL	0	125	250	500	1000	2000	4000	**LC**_**50**_ **, μg/mL**
Mortality%	0.00 ± 0.00	03.33 ± 05.77	10.00 ± 00.00	16.67 ± 05.77	20.00 ± 10.00	30.00 ± 10.00	50.00 ± 10.00	4841.81

At the lowest concentration (125 μg/mL), minimal mortality (3.33 ± 5.77%) was recorded, while lethality reached 50.00 ± 10.00% at the maximum concentration of 4000 μg/mL. Probit analysis of the concentration‐response dataset yielded a calculated median lethal concentration (LC_50_) of 4842 µg/mL (Figure S8). However, this point estimate was accompanied by an exceptionally broad 95% confidence interval (CI) of 1926–518 047 μg/mL. Based on the established toxicity criteria for BSLA (Meyer et al. [74].), where LC_50_ values exceeding 1000 µg/mL are classified as low toxicity, the calculated value for ZnO NPs (A2) indicates low acute toxicity toward *A. salina*. The considerable width of the 95% CI was primarily attributed to the experimental data distribution. Specifically, because 50% mortality was observed at the highest tested concentration (4000 μg/mL), the dataset did not fully capture the upper asymptote of the dose–response curve. This limitation, arising from the absence of data points exhibiting mortality substantially above 50%, reduces the statistical precision of the upper confidence limit of the probit model. Nevertheless, as the LC_50_ point estimate significantly exceeded the 1000 µg/mL threshold, the findings consistently indicate that A2 induces acute lethality only at exceptionally high concentrations. This suggests a low potential for acute environmental risks under the tested conditions, although future assays incorporating higher concentration ranges could further refine the statistical constraints for toxicity estimation in this regard.

### 3.13 Global Benchmarking and Strategic Significance

To evaluate the practical significance and benchmarking of the PPE‐mediated ZnO NPs, the dual‐functional performance of the optimized sample (A2) was compared with a wide array of ZnO‐based materials documented in recent literature (Table 7). This comprehensive comparison, which categorizes materials into biogenic pure oxides, conventional chemical routes, and hybrid composites, provides an extensive overview of the current state‐of‐the‐art in sustainable nanomaterials.

**TABLE 7:** Global comparison of dual‐functional performance (Photocatalysis and Capacitance) of sample A2 with various ZnO‐based materials and synthesis routes.

Material (Source/Method)	***k***_**app**_ **, min^−1^** **MB, 10 ppm**	**Specific capacitance, Fg^−1^** **at 1 A/g**	Reference
ZnO NPs (PPE ‐ A2)	0.065	208.0	**Present work**
ZnO NPs (Starch)	0.064 (pH 7.3)	550.0	[21, 2025]
ZnO NPs (Aloe vera)	n.a.	953.0	[75, 2025]
ZnO NPs (L. nepetifolia)	n.a.	200.0	[76, 2024]
ZnO/rGO composite (L. nepetifolia)	n.a.	667.0	[76, 2024]
ZnO NPs (J. adhatoda)	0.037	n.a.	[77, 2024]
ZnO NPs (Broccoli)	n.a.	31.0	[78, 2025]
ZnO NPs (C. macrocarpa)	0.025	n.a.	[79, 2024]
ZnO (Hydrothermal pure)	n.a.	214.0	[80, 2023]
ZnO (Glycine auto‐combustion)	n.a.	170.0	[81, 2022]
ZnO‐AC (Composite)	n.a.	298.0	[80, 2023]
ZnO (Sol‐gel pure)	0.010	n.a.	[82, 2019]

*Note:* “n.a.” indicates that the parameter was not evaluated or reported in the respective study, highlighting the relative scarcity of dual‐functional reporting in the current biogenic ZnO literature. Values for hybrid composites (ZnO/rGO, ZnO‐AC) utilize conductive carbon scaffolds and are included for contextual reference only; direct quantitative comparison with pure metal oxides is scientifically inappropriate due to fundamentally different electrochemical architectures.

As demonstrated in Table 7, sample A2, synthesized at a strictly ambient temperature of 25°C, achieved a photocatalytic rate constant (*k*_app_ = 0.065 min^−1^) that notably outperformed several biogenic systems, such as J. adhatoda (0.037 min^−1^) [77] and C. macrocarpa (0.025 min^−1^) [79], as well as traditional sol–gel methods (0.010 min^−1^) [82]. Notably, A2 attained the same kinetic efficiency (0.065 min^−1^) at room temperature that our previous starch‐assisted synthesis could only achieve at 80°C (0.064 min^−1^) [21]. This 55°C reduction in the thermal budget, facilitated by the unique phytochemical profile of pomegranate extract, represents a significant advancement in energy‐efficient engineering without compromising catalytic performance. In the field of energy storage, A2 (208 F/g) achieved performance that was competitive with energy‐intensive hydrothermal ZnO (214 F/g) [80], despite a 55°C reduction in synthesis temperature, while clearly outperforming glycine‐based auto‐combustion (170 F/g) [81] and other plant‐mediated systems. The scientific impact of A2 is further underscored by the “Connectivity versus Surface Area” paradox: despite possessing a 38% lower nominal surface area than high‐temperature variants, A2's robust interconnected architecture ensures efficient charge–transfer kinetics (*R*_p_ = 0.0043 Ω), effectively outperforming fragmented morphologies with higher nominal porosity. Although hybrid composites, such as ZnO/rGO (667 F/g) [76] and ZnO‐AC (298 F/g) [80], exhibit higher absolute capacitance owing to their conductive carbon scaffolds, A2 remains remarkably competitive for pure, carbon‐free metal oxides. The defining advantage of our protocol is the “golden compromise” between dual‐functional versatility and environmental safety. While most studies optimize a single property (evidenced by the numerous “n.a.” entries in Table 7), A2 provides a synergistic balance of high ROS generation capacity and pseudocapacitance, maintained alongside negligible acute ecotoxicity (LC_50_ = 4842 μg/mL). This positions PPE‐mediated ZnO as a superior candidate for sustainable deployment in integrated environmental and energy platforms.

## 4 Conclusion

This study established PPE as a highly efficient and sustainable biogenic agent for the green synthesis of ZnO NPs. The results demonstrate that systematic regulation of precursor concentration (0.5–2 M) and synthesis temperature (25°C vs. 80°C) plays a decisive role in the physicochemical evolution of the NPs. Notably, synthesis conducted at ambient temperature with 2 M concentration (sample A2) yielded a robust, interconnected hierarchical architecture that emerged as the optimal material across all evaluated parameters. Sample A2 demonstrated superior dual functionality, achieving an exceptional specific capacitance of 208 F/g and rapid photocatalytic degradation of MB (98% within 60 min). A key finding is that A2 achieved this “golden compromise” by prioritizing morphological interconnectivity over nominal surface area; indeed, although high‐temperature variants such as T2 possessed a 38% higher surface area, their fragmented morphology introduced resistive barriers that hindered performance. This superior structural robustness was validated by high photocatalytic recyclability (90.5% efficiency retained after four cycles) and structural resilience during ultrahigh‐rate electrochemical stress testing (15 A/g), confirming the integrity of the interconnected framework. This optimized performance highlights the efficacy of the PPE‐mediated pathway at room temperature for engineering high‐performance frameworks with enhanced charge separation and optimized defect densities. Furthermore, the eco‐friendly nature of the process was validated by low acute ecotoxicity against *Artemia salina*, confirming the biocompatibility of the resulting NPs. Future research will extend this optimized PPE‐mediated ZnO framework to a broader class of emerging contaminants, such as pharmaceuticals and endocrine disruptors, to further validate its industrial versatility. Overall, this study validates the feasibility of utilizing plant‐based strategies to tailor multifunctional nanomaterials for energy storage and environmental remediation, aligning with the core principles of green chemistry and sustainable nanotechnology.

## Supporting Information

Additional supporting information can be found online in the Supporting Information section.

## Conflicts of Interest

The authors declare no conflicts of interest.

## Supporting information

Supplementary Material

## References

[1] C. Lei , Q. Li , W. Chen , and G. Yu , “Biopolymeric Gels: Advancements in Sustainable Multifunctional Materials,” Advanced Materials 37 (2025): 2419906, 10.1002/adma.202419906. 39924805

[2] D. Behera , P. Priyadarshini , and K. Parida , “ZIF‐8 Metal‐Organic Frameworks and Their Hybrid Materials: Emerging Photocatalysts for Energy and Environmental Applications,” Dalton Transactions 54 (2025): 2681–2708, 10.1039/d4dt02662d. 39810599

[3] M. Salaheldeen , A. Abu‐Dief , and T. El‐Dabea , “Functionalization of Nanomaterials for Energy Storage and Hydrogen Production Applications,” Materials 18 (2025): 768, 10.3390/ma18040768. 40004296 PMC11857648

[4] M. Mahajan , S. Kumar , J. Gaur , et al., “Green Synthesis of ZnO Nanoparticles Using Justicia Adhatodafor Photocatalytic Degradation of Malachite Green and Reduction of 4‐Nitrophenol,” RSC Advances 15, no. 4 (2025): 2958–2980, 10.1039/d4ra08632e. 39881999 PMC11775505

[5] M. S. Yadav , S. M. Bobade , and N. Singh , “Zinc Oxide Nanoparticles and Activated Charcoal‐Based Nanocomposite Electrode for Supercapacitor Application,” Ionics 24, no. 11 (2018): 3611–3630, 10.1007/s11581-018-2527-1.

[6] S. Baruah and J. Dutta , “Hydrothermal Growth of ZnO Nanostructures,“ Science and Technology of Advanced Materials 10, no. 1 (2009): 013001, 10.1088/1468-6996/10/1/013001. 27877250 PMC5109597

[7] A. Sirelkhatim , S. Mahmud , A. Seeni , et al., “Review on Zinc Oxide Nanoparticles: Antibacterial Activity and Toxicity Mechanism,” Nano‐Micro Letters 7, no. 3 (2015): 219–242, 10.1007/s40820-015-0040-x. 30464967 PMC6223899

[8] A. Kołodziejczak‐Radzimska and T. Jesionowski , “Zinc Oxide—from Synthesis to Application: A Review,“ Materials 7, no. 4 (2014): 2833–2881, 10.3390/ma7042833. 28788596 PMC5453364

[9] H. Singh , A. Aldarhami , A. S. Bazaid , et al., “Revisiting the Green Synthesis of Nanoparticles: Uncovering Influences of Plant Extracts as Reducing Agents for Enhanced Synthesis Efficiency and Its Biomedical Applications,” International Journal of Nanomedicine 18 (2023): 4727–4750, 10.2147/ijn.s419369. 37621852 PMC10444627

[10] M. Pirsaheb , U. S. Altimari , T. Gholami , et al., “Green Synthesis of Nanomaterials by Using Plant Extracts as Reducing and Capping Agents,” Environmental Science and Pollution Research 31, no. 17 (2024): 24768–24787, 10.1007/s11356-024-32983-x. 38523214

[11] M. S. S. Danish , M. L. Grilli , A. Mikhaylov , T. Senjyu , L. L. Estrella‐Pajulas , and I. M. Alemaida , “Green Synthesis of Silver Oxide Nanoparticles for Photocatalytic Environmental Remediation and Biomedical Applications,” Metals 12, no. 5 (2022): 769, 10.3390/met12050769.

[12] A. S. Shaban , M. E. Owda , M. M. Basuoni , M. A. Mousa , A. A. Radwan , and A. K. Saleh , “Punica Granatum Peel Extract Mediated Green Synthesis of Zinc Oxide Nanoparticles: Structure and Evaluation of Their Biological Applications,” Biomass Conversion and Biorefinery 14, no. 11 (2022): 12265–12281, 10.1007/s13399-022-03185-7.

[13] A. Verbič , I. Jerman , M. Šala , and M. Gorjanc , “Novel Green In Situ Synthesis of ZnO Nanoparticles on Cotton Using Pomegranate Peel Extract,” Materials 14, no. 16 (2021): 4472, 10.3390/ma14164472. 34442994 PMC8399875

[14] H. M. Ahmed , N. A. Sobhy , W. A. Ibrahem , and M. E. Fawzy , “Green Biosynthesis of Zinc Oxide Nanoparticles Utilizing Pomegranate Peel Extract for Grey Water Treatment,” Solid State Phenomena 342 (2023): 27–36, 10.4028/p-575588.

[15] Q. Zhang , B. You , H. Yuan , K. Ostrikov , M. Xu , and Q. Zhang , “Oxygen Vacancy‐Mediated ZnO Nanoparticle Photocatalyst for Degradation of Methylene Blue,” Applied Sciences 8, no. 3 (2018): 353, 10.3390/app8030353.

[16] A. A. Barzinjy and H. H. Azeez , “Green Synthesis and Characterization of Zinc Oxide Nanoparticles Using Eucalyptus Globulus Labill. Leaf Extract and Zinc Nitrate Hexahydrate Salt,” SN Applied Sciences 2, no. 5 (2020): 991, 10.1007/s42452-020-2813-1.

[17] S. S. A. An , J.‐K. Lee , S. Paek , et al., “Physicochemical Properties of Surface Charge‐Modified ZnO Nanoparticles with Different Particle Sizes,” International Journal of Nanomedicine 9, no. Suppl 2 (2014): 41, 10.2147/ijn.s57923. 25565825 PMC4279853

[18] D. Polsongkram , P. Chamninok , S. Pukird , et al., “Effect of Synthesis Conditions on the Growth of ZnO Nanorods via Hydrothermal Method,“ Physica B: Condensed Matter 403, no. 19‐20 (2008): 3713–3717, 10.1016/j.physb.2008.06.020.

[19] A. Ab Aziz , Z. Khusaimi , and M. Rusop Mahmood , “Effect of Precursor Concentration in the Synthesization of Zno Nanostructures by Solution‐Immersion Method,“ Advanced Materials Research 667 (2013): 501–506, 10.4028/www.scientific.net/AMR.667.501.

[20] H. Takaki , S. Inoue , and Y. Matsumura , “Defects Control in the Synthesis of Low‐Dimensional Zinc Oxide,” Chemical Physics Letters 684 (2017): 113–116, 10.1016/j.cplett.2017.06.050.

[21] R. Djafarou , O. Brahmia , S. Haya , E. Sahmetlioglu , F. KılıçDokan , and T. Hidouri , “Starch‐Assisted Eco‐Friendly Synthesis of Zno Nanoparticles: Enhanced Photocatalytic, Supercapacitive, and Uv‐Driven Antioxidant Properties with Low Cytotoxic Effects,” International Journal of Molecular Sciences 26, no. 2 (2025): 859, 10.3390/ijms26020859. 39859573 PMC11766212

[22] L. He , Z. Tong , Z. Wang , M. Chen , N. Huang , and W. Zhang , “Effects of Calcination Temperature and Heating Rate on the Photocatalytic Properties of ZnO Prepared by Pyrolysis,” Journal of Colloid and Interface Science 509 (2018): 448–456, 10.1016/j.jcis.2017.09.021. 28923742

[23] H. Ahmad Rafaie , N. F. Mohd Yusop , N. F. Azmi , N. S. Abdullah , and N. I. T. Ramli , “Photocatalytic Degradation of Methylene Blue Dye Solution Using Different Amount of ZnO as a Photocatalyst,” Science Letters 15, no. 1 (2021): 11789, 10.24191/sl.v15i1.11789.

[24] V. Cauda , D. Pugliese , N. Garino , et al., “Multi‐Functional Energy Conversion and Storage Electrodes Using Flower‐Like Zinc Oxide Nanostructures,” Energy 65 (2014): 639–646, 10.1016/j.energy.2013.12.025.

[25] H. Savitha , N. Kottam , C. Sampath , G. M. Madhu , and C. S. Aishwarya , “Recent Advances in Cost‐Effective Zno‐Based Electrode Material for Lithium‐Ion Batteries,“ ChemistrySelect 9, no. 37 (2024): e202402489, 10.1002/slct.202402489.

[26] N. Supraja , T. N. V. K. V. Prasad , A. D. Gandhi , D. Anbumani , P. Kavitha , and R. Babujanarthanam , “Synthesis, Characterization and Evaluation of Antimicrobial Efficacy and Brine Shrimp Lethality Assay of Alstoniascholaris Stem Bark Extract Mediated ZnONPs,” Biochemistry and Biophysics Reports 14 (2018): 69–77, 10.1016/j.bbrep.2018.04.004. 29872737 PMC5987000

[27] S. N. A. Mohamad Sukri , K. Shameli , M. Mei‐Theng Wong , S.‐Y. Teow , J. Chew , and N. A. Ismail , “Cytotoxicity and Antibacterial Activities of Plant‐Mediated Synthesized Zinc Oxide (Zno) Nanoparticles Using Punica Granatum (Pomegranate) Fruit Peels Extract,” Journal of Molecular Structure 1189 (2019): 57–65, 10.1016/j.molstruc.2019.04.026.

[28] A. H. Hashem and G. S. El‐Sayyad , “Antimicrobial and Anticancer Activities of Biosynthesized Bimetallic Silver‐Zinc Oxide Nanoparticles (Ag‐Znonps) Using Pomegranate Peel Extract,“ Biomass Conversion and Biorefinery 14, no. 17 (2024): 20345–20357, 10.1007/s13399-023-04126-8.

[29] C. Vivek , B. Balraj , and S. Thangavel , “Structural, Optical and Electrical Behavior of ZnO@Ag Core–shell Nanocomposite Synthesized via Novel Plasmon‐Green Mediated Approach,“ Journal of Materials Science: Materials in Electronics 30, no. 12 (2019): 11220–11230, 10.1007/s10854-019-01467-x.

[30] H. Mohd Yusof , N. Abdul Rahman , R. Mohamad , U. H. Zaidan , and A. A. Samsudin , “Biosynthesis of Zinc Oxide Nanoparticles by Cell‐Biomass and Supernatant of Lactobacillus Plantarum TA4 and Its Antibacterial and Biocompatibility Properties,” Scientific Reports 10, no. 1 (2020): 19996, 10.1038/s41598-020-76402-w. 33204003 PMC7673015

[31] A. Alnehia , A.‐B. Al‐Odayni , A. Al‐Sharabi , A. H. Al‐Hammadi , and W. S. Saeed , “Pomegranate Peel Extract‐Mediated Green Synthesis of Zno‐Nps : Extract Concentration‐Dependent Structure, Optical, and Antibacterial Activity,” Journal of Chemistry 2022 (2022): 1–11, 10.1155/2022/9647793.

[32] A. E. Alprol , A. Eleryan , A. Abouelwafa , A. M. Gad , and T. M. Hamad , “Green Synthesis of Zinc Oxide Nanoparticles Using Padina Pavonica Extract for Efficient Photocatalytic Removal of Methylene Blue,” Scientific Reports 14, no. 1 (2024): 32160, 10.1038/s41598-024-80757-9. 39741157 PMC11688442

[33] K. Dulta , G. KoşarsoyAğçeli , P. Chauhan , R. Jasrotia , and P. K. Chauhan , “Ecofriendly Synthesis of Zinc Oxide Nanoparticles by Carica Papaya Leaf Extract and Their Applications,” Journal of Cluster Science 33, no. 2 (2022): 603–617, 10.1007/s10876-020-01962-w.

[34] S. Maher , S. Nisar , S. M. Aslam , et al., “Synthesis and Characterization of ZnO Nanoparticles Derived from Biomass ( *Sisymbrium Irio*) and Assessment of Potential Anticancer Activity,“ ACS Omega 8, no. 18 (2023): 15920–15931, 10.1021/acsomega.2c07621. 37179630 PMC10173346

[35] A. Fouda , E. Saied , A. M. Eid , et al., “Green Synthesis of Zinc Oxide Nanoparticles Using an Aqueous Extract of Punica Granatum for Antimicrobial and Catalytic Activity,” Journal of Functional Biomaterials 14, no. 4 (2023): 205, 10.3390/jfb14040205. 37103295 PMC10144860

[36] L. YadetaGemachu and A. LealemBirhanu , “Green Synthesis of ZnO, CuO and NiO Nanoparticles Using Neem Leaf Extract and Comparing Their Photocatalytic Activity under Solar Irradiation,” Green Chemistry Letters and Reviews 17, no. 1 (2024): 2293841, 10.1080/17518253.2023.2293841.

[37] T. Asaulyuk , Y. Saribyekova , O. Semeshko , I. Kulish , and Kherson National Technical University , ”Synthesis and Structural Characterization of ZnO Nanoparticles,” Herald of Khmelnytskyi National University. Technical sciences 311 (2022): 35–41, 10.31891/2307-5732-2022-311-4-35-41.

[38] B. L. Cushing , V. L. Kolesnichenko , and C. J. O’Connor , “―Recent Advances in the Liquid‐Phase Syntheses of Inorganic Nanoparticles‖,” Chemical Reviews 104, no. 9 (2004): 3893–3946. 15352782 10.1021/cr030027b

[39] N. T. K. Thanh , N. Maclean , and S. Mahiddine , “―Mechanisms of Nucleation and Growth of Nanoparticles in Solution‖,” Chemical Reviews 114, no. 15 (2014): 7610–7630. 25003956 10.1021/cr400544s

[40] R. Md Akhir , S. Z. Umbaidilah , N. A. Abdullah , et al., “The Potential of Pandanus Amaryllifolius Leaves Extract in Fabrication of Dense and Uniform Zno Microrods,” Micromachines 11, no. 3 (2020): 299, 10.3390/mi11030299. 32182979 PMC7142535

[41] Y. Guan , Q. Hou , and D. Xia , “Effect of Intrinsic Point Defects on ZnO Electronic Structure and Absorption Spectra,“ International Journal of Modern Physics B 34, no. 17 (2020): 2050147, 10.1142/S0217979220501477.

[42] S. Agarwal , L. K. Jangir , K. S. Rathore , M. Kumar , and K. Awasthi , “Morphology‐Dependent Structural and Optical Properties of ZnO Nanostructures,“ Applied Physics A 125, no. 8 (2019): 553, 10.1007/s00339-019-2852-x.

[43] L.‐Y. Wang , B.‐Y. Shi , C.‐B. Yao , et al., “Size and Morphology Modulation in Zno Nanostructures for Nonlinear Optical Applications: A Review,“ ACS Applied Nano Materials 6, no. 12 (2023): 9975–10014, 10.1021/acsanm.3c01509.

[44] R. Maind , S. Halder , A. R. Bhat , et al., “Biomimetic Green Synthesis of ZnO Nanoparticles Using Cheilocostusspeciosus and Gardenia Gummifera with Comprehensive Characterization and Bioactivity Assessment,” Scientific Reports 15 (2025): 44323, 10.1038/s41598-025-30720-z. 41429858 PMC12722286

[45] H. F. Al‐Harbi , M. A. Awad , K. M. O. Ortashi , L. A. AL‐Humaid , A. A. Ibrahim , and A. A. Al‐Huqail , “Green Synthesis of Zinc Oxide Nanoparticles: Physicochemical Characterization, Photocatalytic Performance, and Evaluation of Their Impact on Seed Germination Parameters in Crops,” Catalysts 15, no. 10 (2025): 924, 10.3390/catal15100924.

[46] V. Y. Kolotygina , A. Y. Zhilyakov , M. A. Bukharinova , E. I. Khamzina , and N. Y. Stozhko , “Green Synthesis of ZnO Nanoparticles: Effect of Synthesis Conditions on Their Size and Photocatalytic Activity,” ChemEngineering 10, no. 1 (2026): 15, 10.3390/chemengineering10010015.

[47] X. Wang , Y. Zhang , C. Hao , F. Feng , H. Yin , and N. Si , “Solid‐Phase Synthesis of Mesoporous Zno Using Lignin‐Amine Template and Its Photocatalytic Properties,“ Industrial & Engineering Chemistry Research 53, no. 16 (2014): 6585–6592, 10.1021/ie404179f.

[48] J. Liu , F. Gao , L. Wu , et al., “Size Effect on Oxygen Vacancy Formation and Gaseous Adsorption in ZnOnanocrystallites for Gas Sensors: A First Principle Calculation Study,” Applied Physics A 126, no. 6 (2020): 454, 10.1007/s00339-020-03643-x.

[49] H. Wang and Y. Chiang , “Thermodynamic Stability of Intergranular Amorphous Films in Bismuth‐Doped Zinc Oxide,” Journal of the American Ceramic Society 81, no. 1 (1998): 89–96, 10.1111/j.1151-2916.1998.tb02299.x.

[50] H. Tamura , K. Mita , A. Tanaka , and M. Ito , “Mechanism of Hydroxylation of Metal Oxide Surfaces,” Journal of Colloid and Interface Science 243, no. 1 (2001): 202–207, 10.1006/jcis.2001.7864.

[51] W. W. Wright , M. Laberge , and J. M. Vanderkooi , “Surface of Cytochrome *c*: Infrared Spectroscopy of Carboxyl Groups,” Biochemistry 36, no. 48 (1997): 14724–14732, 10.1021/bi971559n. 9398192

[52] P. L. Meena , A. K. Surela , L. K. Chhachhia , J. Meena , and R. Meena , “Investigation of the Photocatalytic Potential of C/N‐Co‐Doped ZnO Nanorods Produced *via* a Mechano‐Thermal Process,“ Nanoscale Advances 7, no. 5 (2025): 1335–1352, 10.1039/D4NA00890A. 39839223 PMC11744485

[53] N. Seeram , L. Adams , S. Henning , et al., “In Vitro Antiproliferative, Apoptotic and Antioxidant Activities of Punicalagin, Ellagic Acid and a Total Pomegranate Tannin Extract Are Enhanced in Combination with Other Polyphenols as Found in Pomegranate Juice,” The Journal of Nutritional Biochemistry 16, no. 6 (2005): 360–367, 10.1016/j.jnutbio.2005.01.006. 15936648

[54] A. H. Hashem , A. A. Al‐Askar , M. R. Saeb , K. A. Abd‐Elsalam , A. S. El‐Hawary , and M. S. Hasanin , “Sustainable Biosynthesized Bimetallic ZnO@SeO Nanoparticles from Pomegranate Peel Extracts: Antibacterial, Antifungal and Anticancer Activities,” RSC Advances 13, no. 33 (2023): 22918–22927, 10.1039/D3RA03260D. 37520090 PMC10377119

[55] H. M. Abdelmigid , N. A. Hussien , A. A. Alyamani , M. M. Morsi , N. M. AlSufyani , and H. A. Kadi , “Green Synthesis of Zinc Oxide Nanoparticles Using Pomegranate Fruit Peel and Solid Coffee Grounds vs. Chemical Method of Synthesis, with Their Biocompatibility and Antibacterial Properties Investigation,“ Molecules 27, no. 4 (2022): 1236, 10.3390/molecules27041236. 35209025 PMC8877600

[56] A. K. Khan , S. Renouard , S. Drouet , et al., “Effect of Uv Irradiation (A and c) on Casuarina Equisetifolia‐Mediated Biosynthesis and Characterization of Antimicrobial and Anticancer Activity of Biocompatible Zinc Oxide Nanoparticles,” Pharmaceutics 13, no. 11 (2021): 1977, 10.3390/pharmaceutics13111977. 34834392 PMC8622962

[57] A. B. Djurišić , Y. H. Leung , K. H. Tam , et al., “Defect Emissions in ZnO Nanostructures,” Nanotechnology 18, no. 9 (2007): 095702, 10.1088/0957-4484/18/9/095702.

[58] Y. M. Hunge , A. A. Yadav , and B. M. Mohite , “Basics of Photocatalysis and Different Strategy for Enhancing the Photocatalytic Efficiency,” American Journal of Engineering and Applied Sciences 13, no. 2 (2020): 265–268, 10.3844/ajeassp.2020.265.268.

[59] J.‐M. Herrmann , “Heterogeneous Photocatalysis: Fundamentals and Applications to the Removal of Various Types of Aqueous Pollutants,“ Catalysis Today 53, no. 1 (1999): 115–129, 10.1016/S0920-5861(99)00107-8.

[60] J. Schneider , M. Matsuoka , M. Takeuchi , et al., “Understanding tio_2_ Photocatalysis : Mechanisms and Materials,” Chemical Reviews 114, no. 19 (2014): 9919–9986, 10.1021/cr5001892N. 25234429

[61] X. Wang , S. Han , Q. Zhang , N. Zhang , and D. Zhao , “Photocatalytic Oxidation Degradation Mechanism Study of Methylene Blue Dye Waste Water with GR/iTO_2_ ,” MATEC Web of Conferences 238 (2018): 03006, 10.1051/matecconf/201823803006.

[62] M. R. Hoffmann , S. T. Martin , W. Choi , and D. W. Bahnemann , “Environmental Applications of Semiconductor Photocatalysis,” Chemical Reviews 95, no. 1 (1995): 69–96, 10.1021/cr00033a004.

[63] G. G. Welegergs , H. G. Gebretinsae , R. Akoba , N. Matinsie , Z. Y. Nuru , and M. Maaza , “Electrochemical Properties of Green Synthesised Zinc Oxide (Zno) Nanoparticles,“ MRS Advances 5, no. 21‐22 (2020): 1103–1112, 10.1557/adv.2020.119.

[64] M. Thejaswini , V. Lakshmi Ranganatha , H. B. Vasanth Patil , S. Pramila , G. Nagaraju , and C. Mallikarjunaswamy , “Phyto‐Mediated Facile Synthesis of ZnO Nanoparticles: Enhanced Photocatalysis, Biological, and Electrochemical Properties,“ Ionics 30, no. 10 (2024): 6611–6629, 10.1007/s11581-024-05710-2.

[65] A. J. Bard and L. R. Faulkner , Electrochemical Methods: Fundamentals and Applications, 2nd ed., (Wiley, 2001).

[66] B. E. Conway , Electrochemical Supercapacitors: Scientific Fundamentals and Technological Applications, 1st ed., (Springer, 1999).

[67] H. Pecenek , F. K. Dokan , M. S. Onses , E. Yılmaz , and E. Sahmetlioglu , “High Energy Density Hybrid Supercapacitors Based on Graphitic Carbon Nitride Modified BiFeO_3_ and Biomass‐Derived Activated Carbon,” Journal of Energy Storage 64 (2023): 107075, 10.1016/j.est.2023.107075.

[68] A. S. Aricò , P. Bruce , B. Scrosati , J.‐M. Tarascon , and W. Van Schalkwijk , “Nanostructured Materials for Advanced Energy Conversion and Storage Devices,” Nature Materials 4, no. 5 (2005): 366–377, 10.1038/nmat1368. 15867920

[69] S. Sivakumar , Y. Robinson , and N. A. Mala , “Pseudocapacitancebehavior of Copper and Nickel Co‐Doped Zinc Oxide Nanoparticles with Enhanced Photocatalytic Performance,” Journal of Materials Science: Materials in Electronics 34, no. 11 (2023): 1–21, 10.1007/s10854-023-10427-5.

[70] V. Augustyn , P. Simon , and B. Dunn , “Pseudocapacitive Oxide Materials for High‐Rate Electrochemical Energy Storage,” Energy & Environmental Science 7, no. 5 (2014): 1597, 10.1039/c3ee44164d.

[71] M. E. Orazem and B. Tribollet , Electrochemical Impedance Spectroscopy (Wiley, 2008).

[72] B. E. Conway , “Transition from “Supercapacitor” to “Battery” Behavior in Electrochemical Energy Storage,” Journal of the Electrochemical Society 138, no. 6 (1991): 1539–1548, 10.1149/1.2085829.

[73] P. Simon and Y. Gogotsi , “Materials for Electrochemical Capacitors,“ Nature Materials 7, no. 11 (2008): 845–854, 10.1038/nmat2297. 18956000

[74] B. Meyer , N. Ferrigni , J. Putnam , L. Jacobsen , D. Nichols , and J. McLaughlin , “Brine Shrimp: A Convenient General Bioassay for Active Plant Constituents,“ Planta Medica 45, no. 05 (1982): 31–34, 10.1055/s-2007-971236. 7100305

[75] M. Bhatt , K. Gautam , A. Verma , and A. K. Sinha , “Structural, Optical, Surface Chemical, and Electrochemical Characterization of Aloe Vera‐Assisted ZnO Nanostructures for Supercapattery Applications,” Materials Advances 6, no. 16 (2025): 5618–5632, 10.1039/d5ma00556f. PMC12377184 40861976

[76] J. Meena , G. Pavithra , D. Anusha , A. S. Kumar , and K. Santhakumar , “The Green Approach of ZnO NPs and Its Antioxidant, Hemolytic, and Photocatalytic Activity and Functionalized r‐GO‐ZnO for Energy Storage Application,” Journal of Materials Science: Materials in Electronics 34 (2023): 1131, 10.1007/s10854-023-10373-2.

[77] A. B. Anik , M. Hossen Akash , M. A. Alam , M. Z. Alam , D. Sarker , and N. Sultana , “Green Synthesis of ZnO Nanoparticles Using Justicia Adhatoda for Effective Photocatalytic Degradation of Methylene Blue Dye,” RSC Advances 15 (2025): 45874–45888, 10.1039/D5RA06656E. 41293286 PMC12641247

[78] S. Ersoy , M. H. Aleinawi , E. Erdem , F. Kaya , C. Kaya , and B. Koc , “Green Synthesized of Zinc Oxide Nanoparticles and Calcined Biowaste for Sustainable Electrodes to Asymmetric Supercapacitors,” Scientific Reports 15 (2025): 40087, 10.1038/s41598-025-23895-y. 41249253 PMC12624125

[79] A. B. Siddique , M. A. Shaheen , A. Abbas , et al., “Thermodynamic and Kinetic Insights into Azo Dyes Photocatalytic Degradation on Biogenically Synthesized ZnO Nanoparticles and Their Antibacterial Potential,” Heliyon 10, no. 23 (2024): e40679, 10.1016/j.heliyon.2024.e40679. 39717568 PMC11665338

[80] K. G. Manish , K. Yogesh , S. Neeleshwar , and K. S. Sanjay , “Hydrothermally Grown Zinc Oxide Nanostructures@Carbon Composites for Supercapacitor Application,” physica status solidi (a) 220, no. 1 (2022): 2200451, 10.1002/pssa.202200451.

[81] A. AlFawaz , A. Ahmad , N. Ahmad , and F. A. Alharthi , “Glycine Based Auto‐Combustion Synthesis of ZnO Nanoparticles as Electrode Material for Supercapacitor,” Physica Scripta 97, no. 3 (2022): 030009, 10.1088/1402-4896/ac52f8.

[82] K. A. Isai and V. S. Shrivastava , “Photocatalytic Degradation of Methylene Blue Using ZnO and 2%Fe–ZnO Semiconductor Nanomaterials Synthesized by Sol–gel Method: A Comparative Study,” SN Applied Sciences 1 (2019): 1247, 10.1007/s42452-019-1279-5.

