# Supplementary material for: Dual‐Functional and Low‐Toxicity ZnO Nanoparticles From Pomegranate Peel: Impact of Synthesis Parameters on Photocatalytic and Supercapacitive Performance
Source: ChemistryOpen. 2026 Apr 21;15(5):e70209. doi: 10.1002/open.70209 (PMC13098670; doi:10.1002/open.70209)
Supplement: Supplementary file 1 — Supplementary Material [file OPEN-15-e70209-s001.pdf]

Supporting Information

Dual-Functional and Low-Toxicity ZnO Nanoparticles from Pomegranate Peel: Impact of Synthesis Parameters on Photocatalytic and Supercapacitive Performance

Roumaissa Djafarou[1], Ouarda Brahmia[1], Naima Benchikha[2], Fatma Kılıç Dokan[3], ErtugrulSahmetlioglu[4], Ayomide Victor Atoki[5]*, Mohammed Messaoudi[6]

[1] Laboratoire des Techniques Innovantes de Préservation de l'Environnement, Université de Constantine 1, Constantine 25000, Algeria, roumaissa.djafarou@student.umc.edu.dz (R.D.); ouarda.brahmia@umc.edu.dz (O.B.)

[2] Laboratory of Applied Chemistry and Environment (LCAE), Department of Chemistry, Faculty of Exact Sciences, University of Hamma Lakhdar, P.O. Box 789, El-Oued 39000, Algeria, benchikha-naima@univ-eloued.dz

[3] Department of Chemistry and Chemical Processing Technologies, Mustafa Çıkrıkcıoglu Vocational School, Kayseri University, Kayseri 38039, Turkey, fatmakilic@kayseri.edu.tr

[4] Department of Basic Sciences of Engineering, Kayseri University, Kayseri 38039, Turkey, sahmetlioglu@kayseri.edu.tr

[5]Department of Biochemistry, Kampala International University, Ishaka, Uganda

[6]Laboratory of Research on Bioactive Products and Biomass Valorization, Department of Chemistry, Higher Normal School of Kouba (ENS), P.O. Box 92, Vieux-Kouba, Algiers 16308, Algeria.

* Correspondences atokiav@kiu.ac.ug

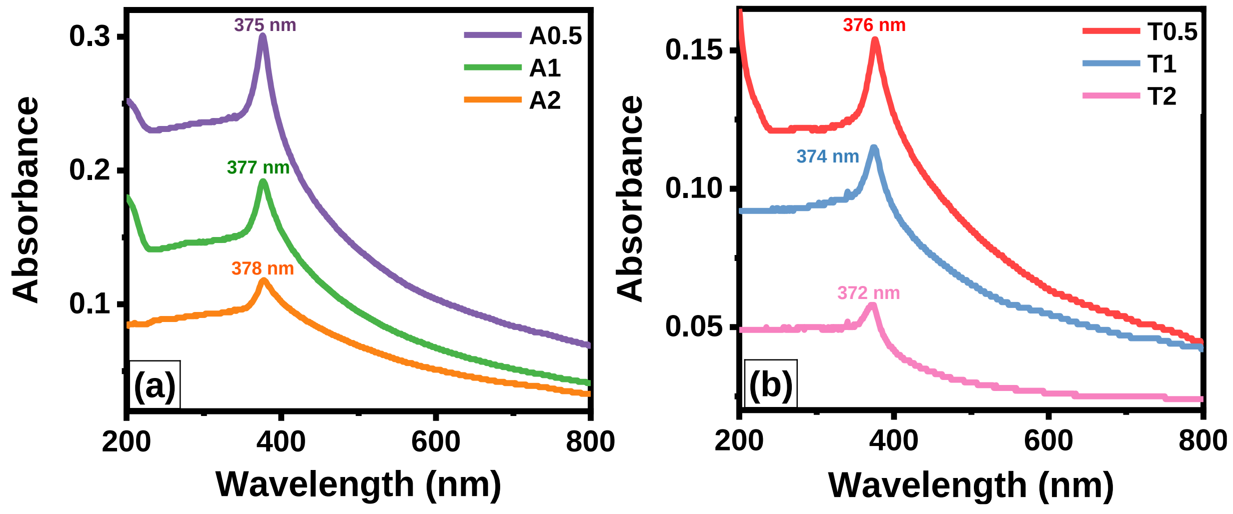

Figure S1. UV–Visible absorbance spectra for ZnO samples synthesized at (a) 25 °C (A-series) and (b) 80 °C (T-series).

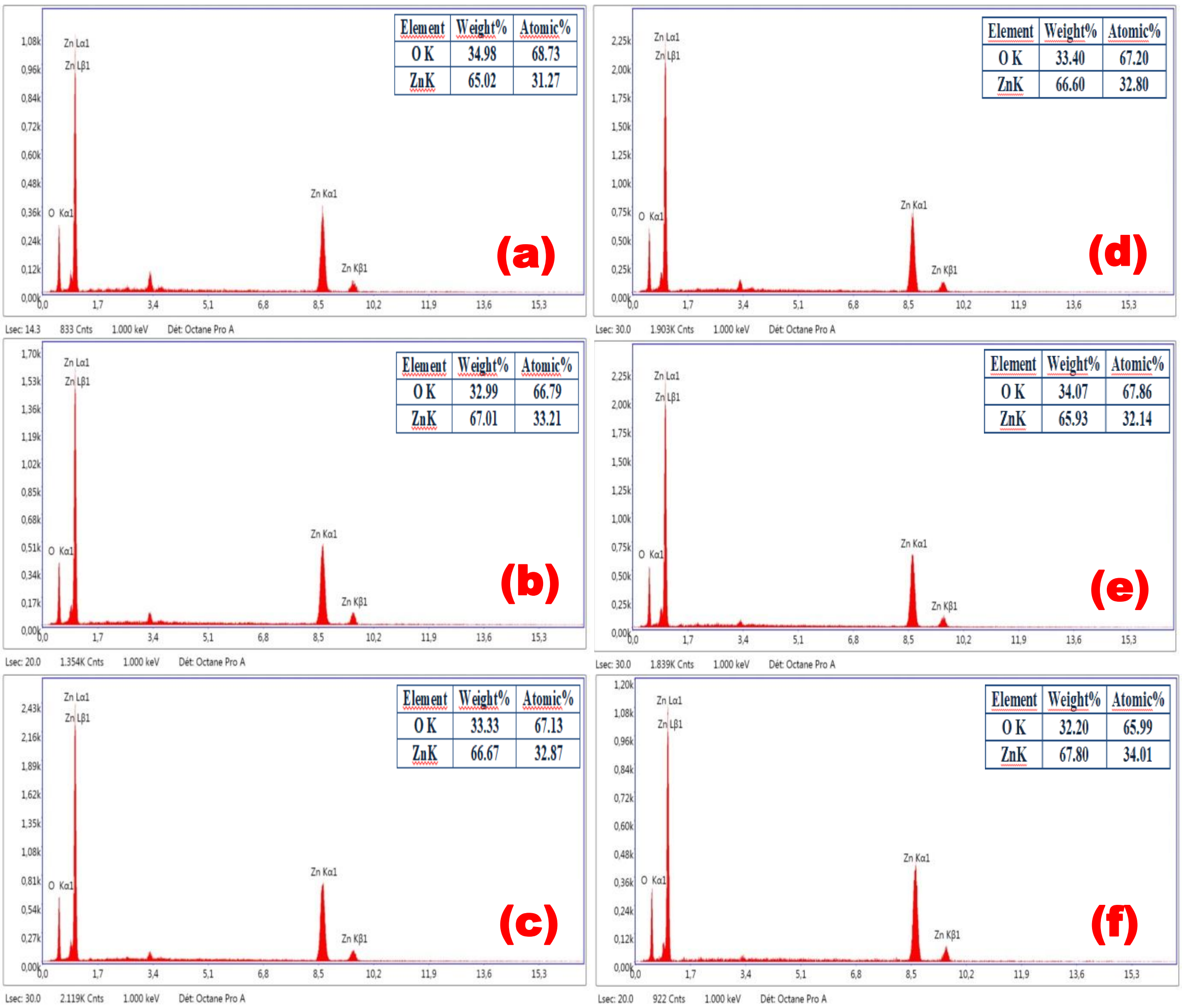

Figure S2: (a-f) Graphs of Energy Dispersive Spectroscopy (EDS) and elemental weight and atomic percentages of all synthesized materials A0.5, A1, A2, T0.5, T1 and T2

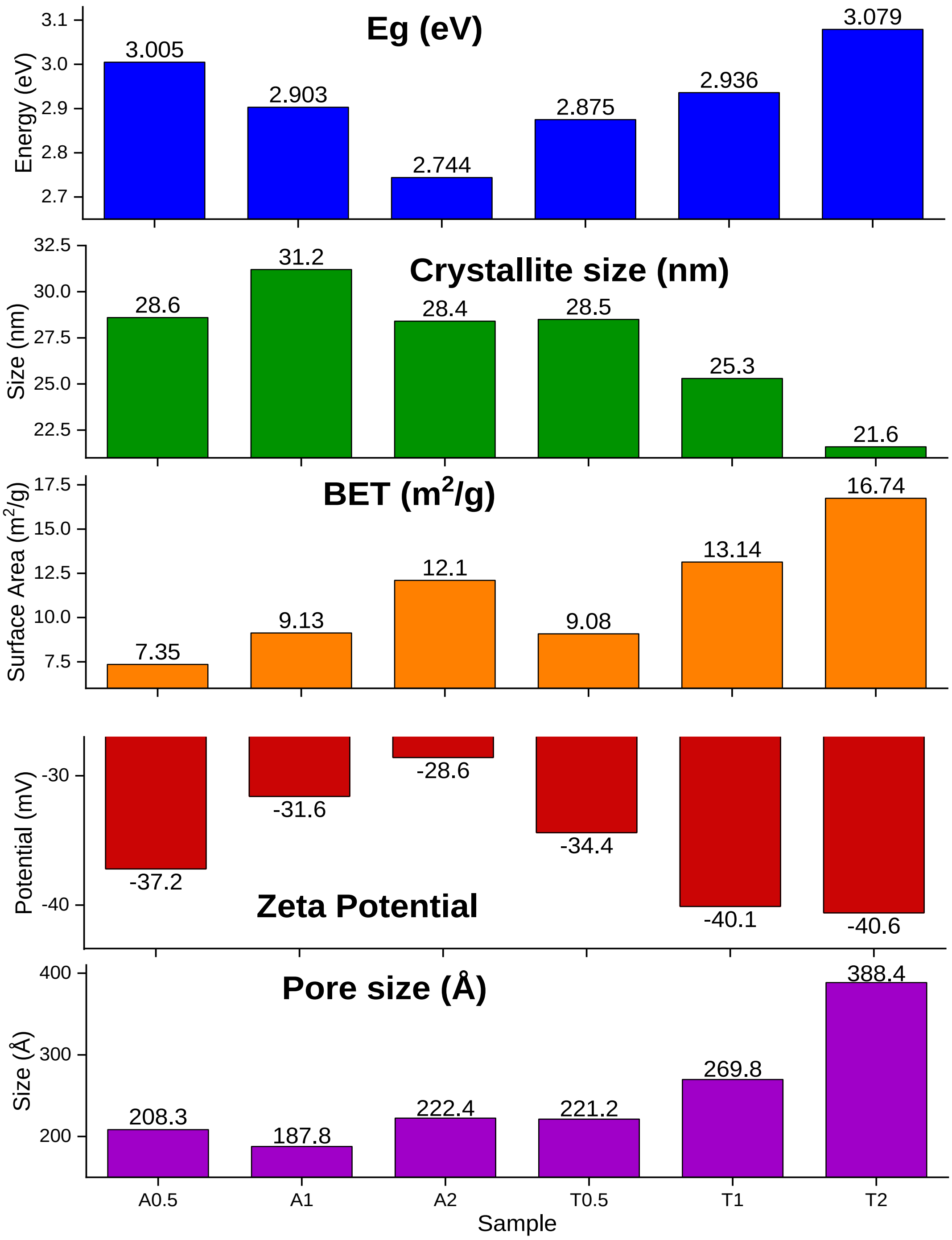

Figure S3: Evolution of Structural and Electronic Properties of ZnO NPs as a Function of Synthesis Temperature and Precursor Concentration

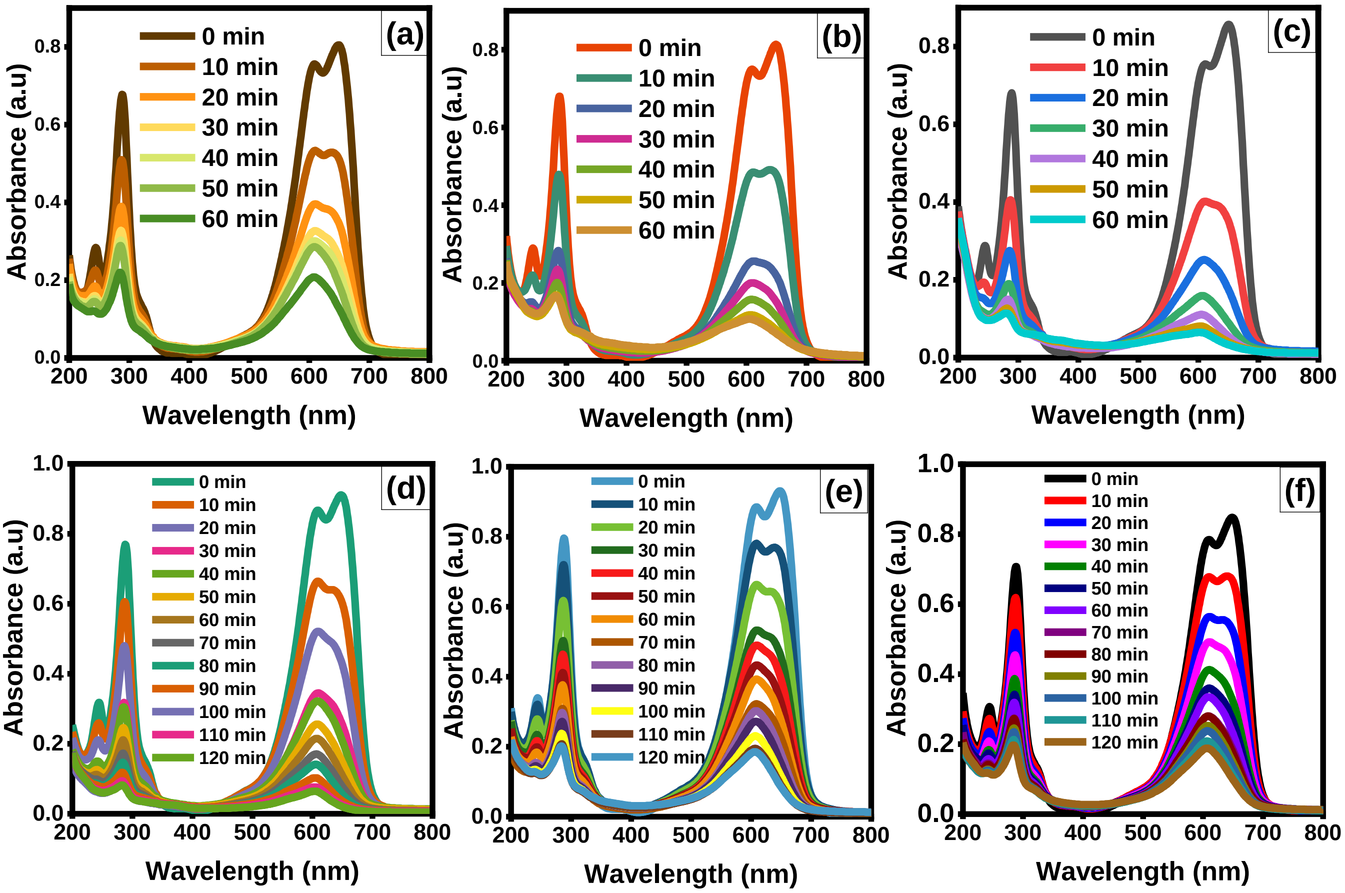

Figure S4: Temporal evolution of UV-Vis spectra of MB progressively photodegraded by synthesized ZnO NPs: (a) A0.5, (b) A1, (c) A2, (d) T0.5, (e) T1, (f) T2

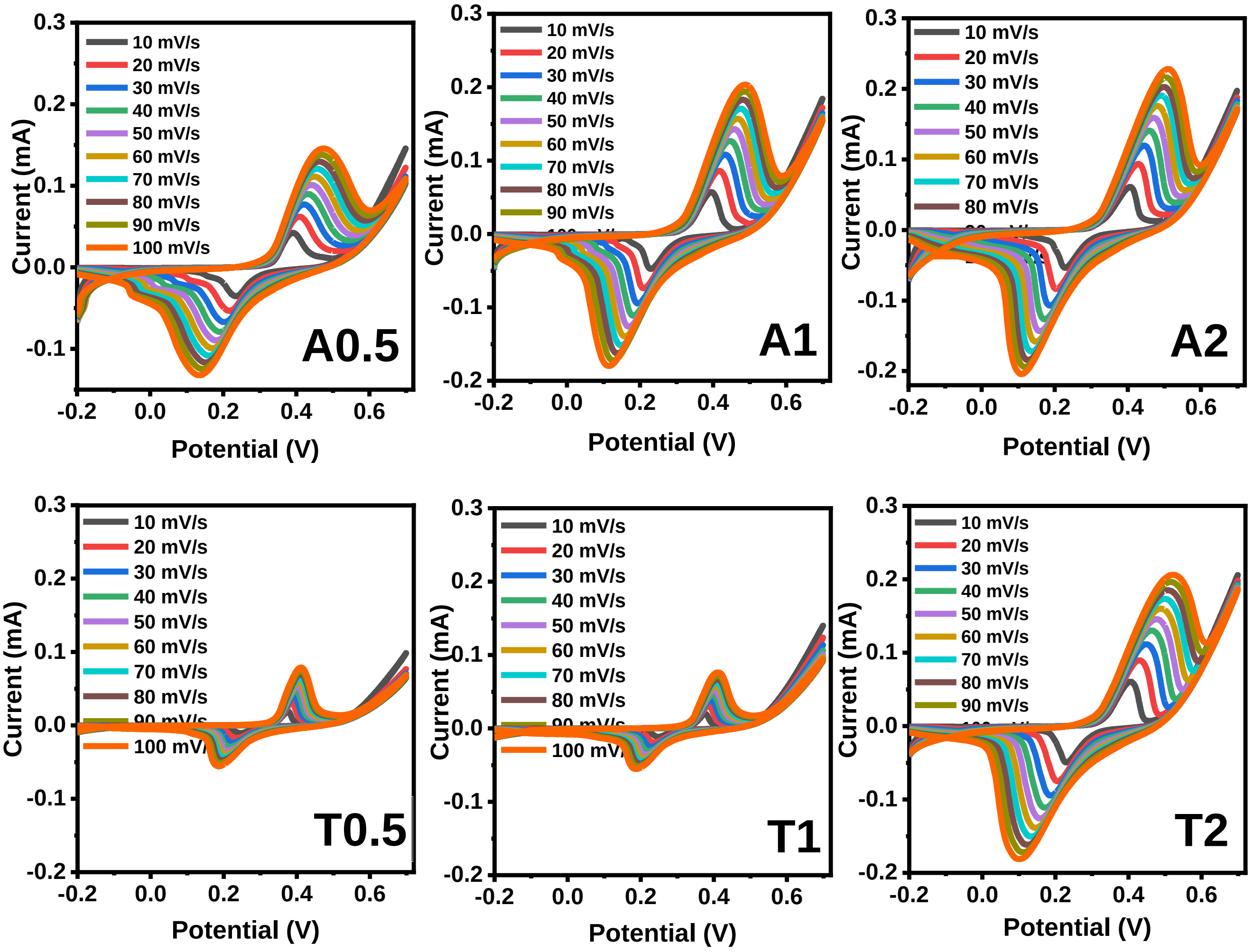

Figure S5: CV curves of the different synthesized ZnO NPs at different scan rates

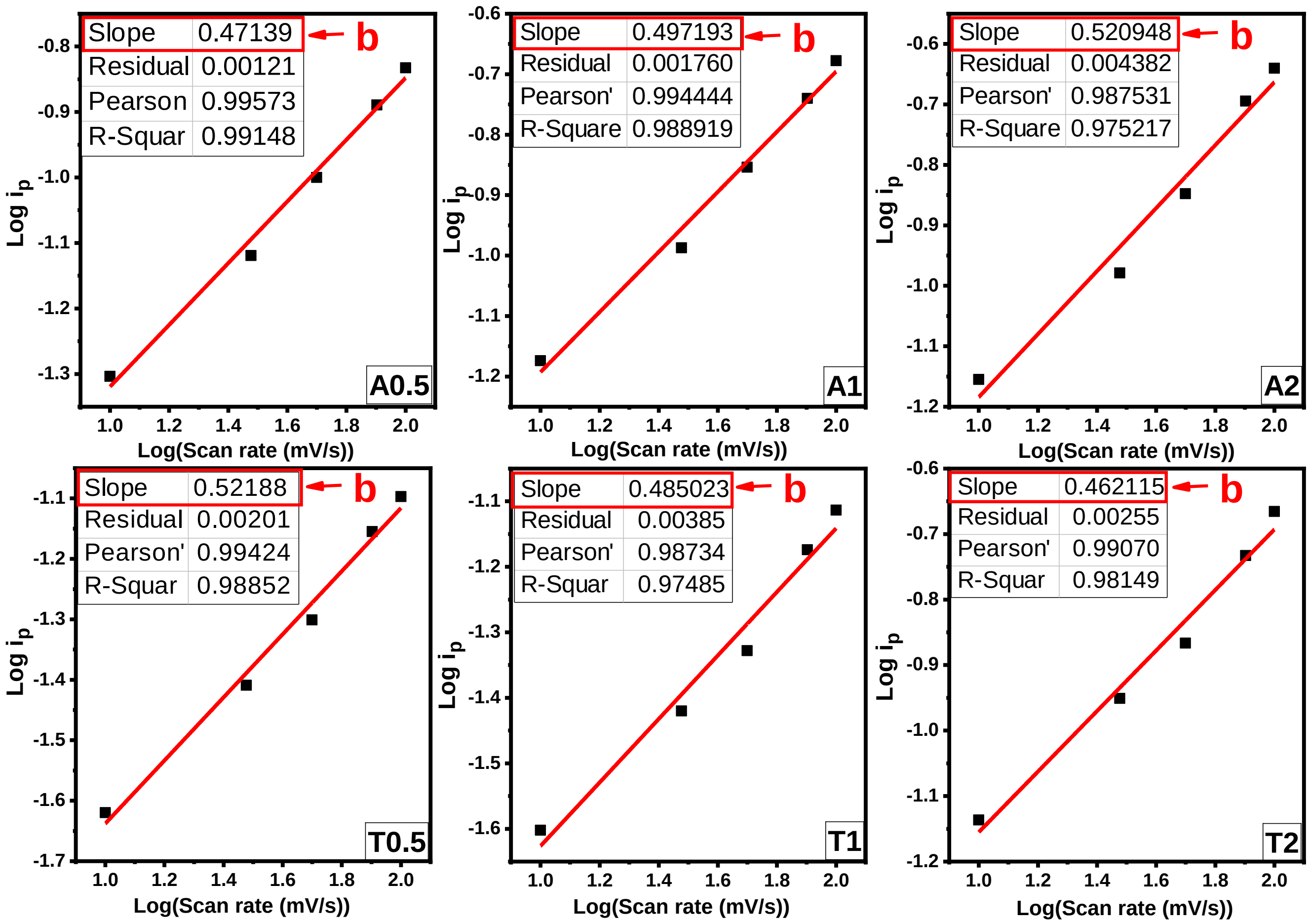

Figure S6: Power-law analysis (logi_p versus logv) for the determination of *b*-values for samples A0.5, A1, A2, T0.5, T1, and T2.

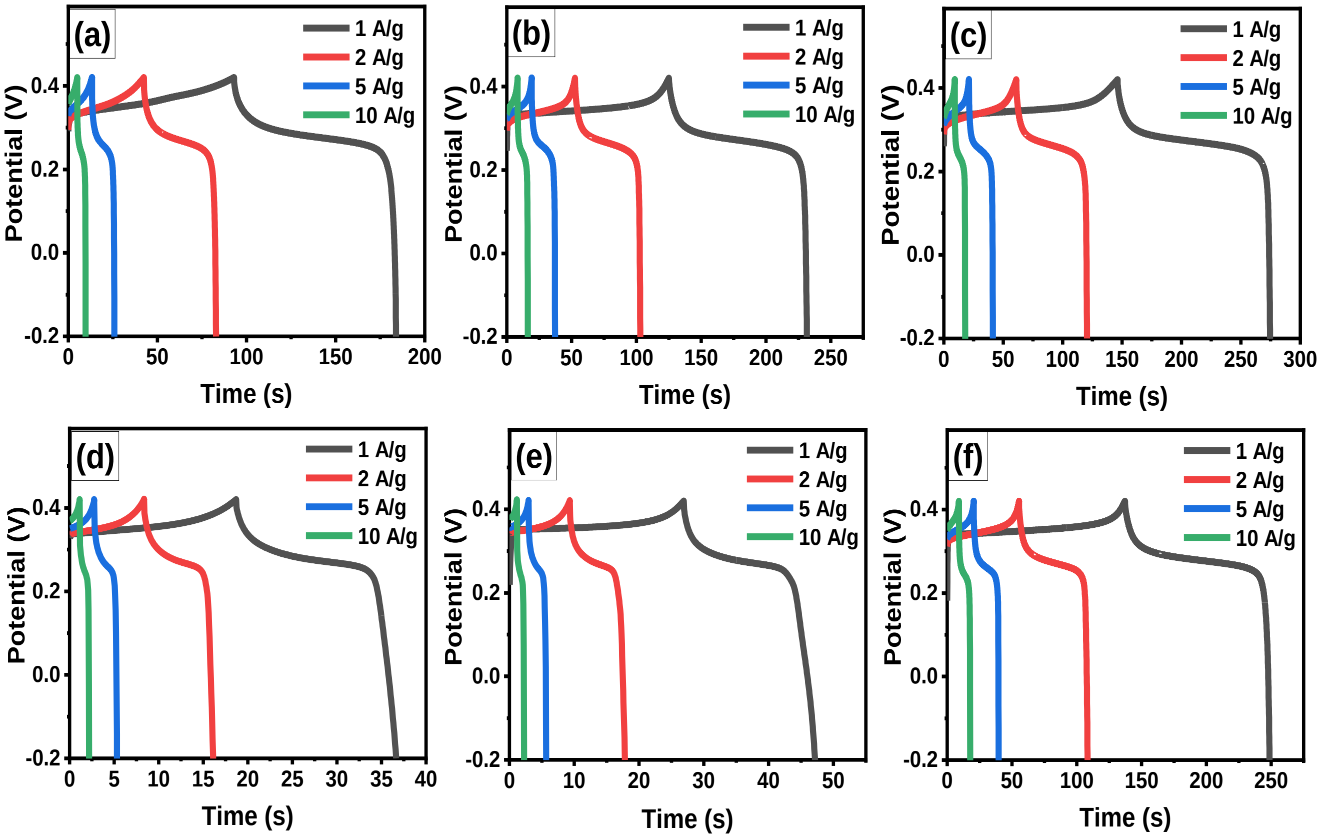

Figure S7: GCD curves of the ZnO electrode materials at different current densities: (a) A0.5, (b) A1, (c) A2, (d) T0.5, (e) T1, and (f) T2

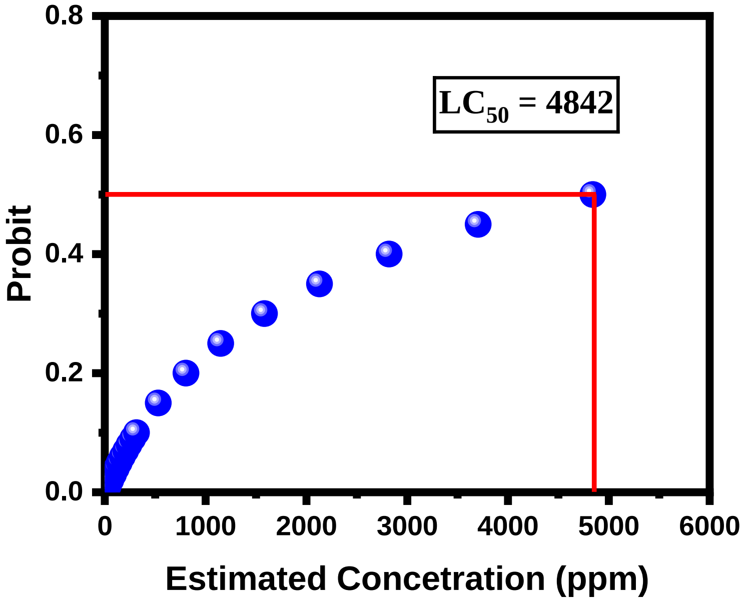

Figure S8: Probit analysis of the mortality-concentration response for LC_{50} determination
